# Improving Spectral Similarity and Molecular Network Reliability through Noise Signal Filtering in MS/MS Spectra

**DOI:** 10.1021/acs.analchem.5c02109

**Published:** 2025-07-17

**Authors:** Nicola Dalla Valle, Mar Garcia-Aloy, Peter Robatscher, Pietro Franceschi, Michael Oberhuber

**Affiliations:** † 19034 University of Trento, Trento, TN 38100, Italy; ‡ Research Innovation Centre, Fondazione Edmund Mach, San Michele a/A, TN 38010, Italy; § Laimburg Research Centre, Laimburg 6 − Pfatten (Vadena), Auer (Ora), BZ 39040, Italy

## Abstract

In mass spectrometry, fragmentation spectra play a central role in compound identification. However, noise in MS/MS spectra can significantly impact similarity scores and molecular network (MN) reliability, leading to inaccurate compound annotation in untargeted metabolomics. This work investigates the influence of noise on MS/MS similarity scores and molecular network structure. Noise elimination increased similarity scores for homologous spectra, enhancing match affordability. In MNs, effective noise management improved network structure, resulting in more interpretable networks with fewer edges and enhanced clustering, decreasing false-positive connections. To quantitatively assess these improvements, a minimum spanning tree (MST) analysis was performed, revealing denser regions in the denoised MNs. An increasing cutoff of noise threshold can lead to an overlay between two or more different compound spectra. A data-specific workflow was developed to identify the optimal threshold for denoising, balancing spectra quality and network integrity during noise elimination, by incorporating statistics calculated on the distribution of the MST distances and the number of fragment ions, which could be explained by an in-silico fragmentation algorithm. Finally, a faster-tailored denoising method, based solely on the intensity of individual spectral ions, demonstrated performance comparable to the previously cited fixed threshold approaches.

## Graphical abstract

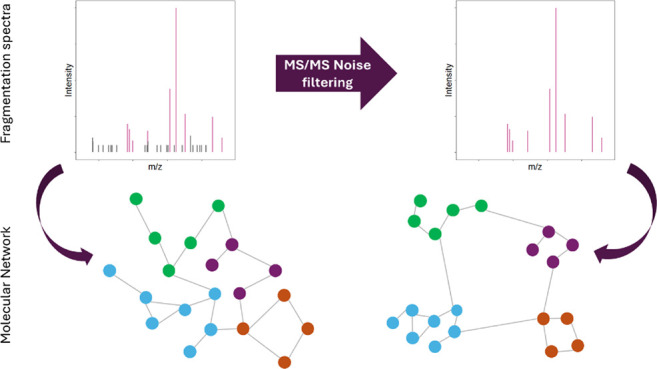

## Introduction

Untargeted metabolomics aims to analyze the widest possible array of compounds within a sample without relying on a predefined list of molecules, positioning it as a hypothesis-generating approach. Mass spectrometry (MS) has emerged as one of the primary analytical tools for the detection, annotation, and relative quantification of small molecules in complex matrices, offering high sensitivity in detecting diverse molecular species.^1,2^ However, the major limitation in the field of untargeted metabolomics results from the fact that unambiguous chemical identification cannot be based on the measurement of the mass-to-charge ratio (*m*/*z*) of the individual ions alone. This fundamental limitation can be, at least partially, overcome by tandem mass spectrometry (MS/MS), which enables to obtain fragmentation spectra from selected ions, providing additional structural information which can support molecular identification. Despite the improvements in MS performance, however, identification still represents the bottleneck for untargeted metabolomics studies.

Traditionally, the identification of chemical compounds is based on the comparison of experimental MS/MS spectra with in-house or online shared libraries of analytical standards.^3^ In addition, molecular networking has been increasingly used to organize and identify ions in large MS/MS data sets.^2^

Molecular networking (MN) is a computational technique that groups MS/MS spectra according to their spectral similarity, revealing relationships between molecules which help during the identification process. The founding assumption behind MN is that structurally similar molecules will generate similar MS/MS spectra.^4,5^ In this context, MS/MS similarity is then a central metric since it serves as a quantitative proxy of structural similarity. Several functions to calculate similarity scores have been proposed for spectral comparison (e.g., Pearson’s *r*, Spearman’s rho, dot-product and Cosine score), with the objective of better separating similar MS/MS spectra from dissimilar ones.^6^ They mostly rely on comparing the intensity of shared *m*/*z* values between fragmentation spectra. Global Natural Products Social Molecular Networking (GNPS), a widely used web-based platform for MN MS/MS data analysis,^4^ implements similarity score calculation taking into consideration also the offset by the same *m*/*z* difference as the precursor ion (i.e., neutral losses).^2^

As it can be easily expected, low-quality spectra can mislead meaningful spectral relationships, reducing annotation ability and molecular networks’ accuracy and robustness. In particular, the presence of background noise is one of the factors that typically impact the quality of fragmentation spectra, since it results in “false” matches among the *m*/*z* values of the two spectra. MS instruments can indeed produce noise that can occur at any retention time and *m*/*z*.^7^

The improvement of the quality of fragmentation spectra is hence of pivotal importance to foster the annotation process. The most straightforward way to improve the spectral quality relies on the introduction of robust experimental workflows and the optimization of technical and instrumental parameters.^8^ However, postacquisition computational data processing routines represent a further step in the production of high-quality MS/MS spectra and related MN analysis.^9^

One of the most commonly used filtering methods for noise reduction in MS/MS spectra is the 5% intensity cutoff based on the base peak intensity.^10^ Also the generation of consensus spectra, by clustering MS/MS scans for the same precursor and averaging or selecting a representative spectrum, can reduce random noise and improve spectral quality.^11^ Several alternative workflows have been proposed to improve fragmentation spectra quality in different contexts.^12–16^ Despite the availability of various denoising methods, relying on different approaches and assumptions, to the best of our knowledge, the impact of denoising on similarity scores and MN clarity has not been extensively investigated.

In this study, we evaluate the impact of noise on similarity score and investigate its effect on homologous spectra matches. We then analyze the effect of noise elimination on MN structure, using both reference standards libraries and data from a biological sample case study. Finally, we propose a workflow to identify the optimal data-dependent noise cutoff threshold, validated by applying a newly developed tailored cleaning method.

## Experimental Section

### Data Acquisition for In-House Standards Library

A series of different analytical standards were analyzed using an Orbitrap LTQ-XL (Thermo Fisher, Bremen, Germany) operating in data-dependent acquisition mode. Fragmentation spectra were generated using collision-induced dissociation at voltages between 35 and 45 V. In this study, only spectra corresponding to [M – H]^−^ and [M + H]^+^ ions of the corresponding standards were selected. Furthermore, we retained only those spectra for which the nominal “precursorMz” value was equal to the nominal “isolationWindowTargetMz”, as well as those spectra with entropy values below 3, as poor-quality noisy spectra are characterized by higher entropy levels.^17^ Table S1 lists the compounds considered with the chromatographic method used for their acquisition.

### External Library MS/MS Data

GNPS fragmentation spectra were downloaded from the MassBank of North America (MoNA) website (in May 2025). Only MS/MS spectra generated from [M + H]^+^ and [M – H]^−^ adducts containing more than 20 ions were considered. For each spectrum, the number of structurally explained peaks was calculated using the SIRIUS software, as described in the section “Identification of MS/MS explained ions”. In order to be able to evaluate the effect of noise removal, only those spectra with a ratio of SIRIUS explained ions to total ions less than 0.75 were considered, otherwise it was considered that the spectra were already denoised or manually curated. Furthermore, to optimize computational efficiency, spectra containing fewer than 1000 ions were selected. The data were then categorized into “Orbitrap” and “Tof” groups based on the acquisition instrument reported.

### Biological Sample Data

MS/MS data were obtained from a biological data set,^18^ available on the MetaboLights platform (study identifier: MTBLS5261). For each identified compound (Table S3), the fragmentation spectrum with the lowest entropy was selected based on matching of retention time (within a 10-s tolerance) and precursor mass (within a 0.001 Da tolerance). In cases where spectral selection was ambiguous, spectra were manually selected from the raw data according to the MS/MS spectra provided in the Supporting Information of the original publication.^18^

### Tailored Noise-Cleaning Procedure

Based on the MS/MS data structure, we developed a method to filter the noise ions in MS/MS spectra relying only on their intensity. This approach applies to individual spectra regardless of the compound’s nature, structure, or precursor intensity. Figure 1 illustrates the rationale behind the filtering approach based on the assumption that the intensity of the uninformative noise (e.g., electronic noise) follows a uniform distribution while ions generated from “true” molecular fragmentation show a larger intensity that deviates from this background distribution.^19^ For each MS/MS spectra the following steps were applied for noise management:

**Figure 1:**
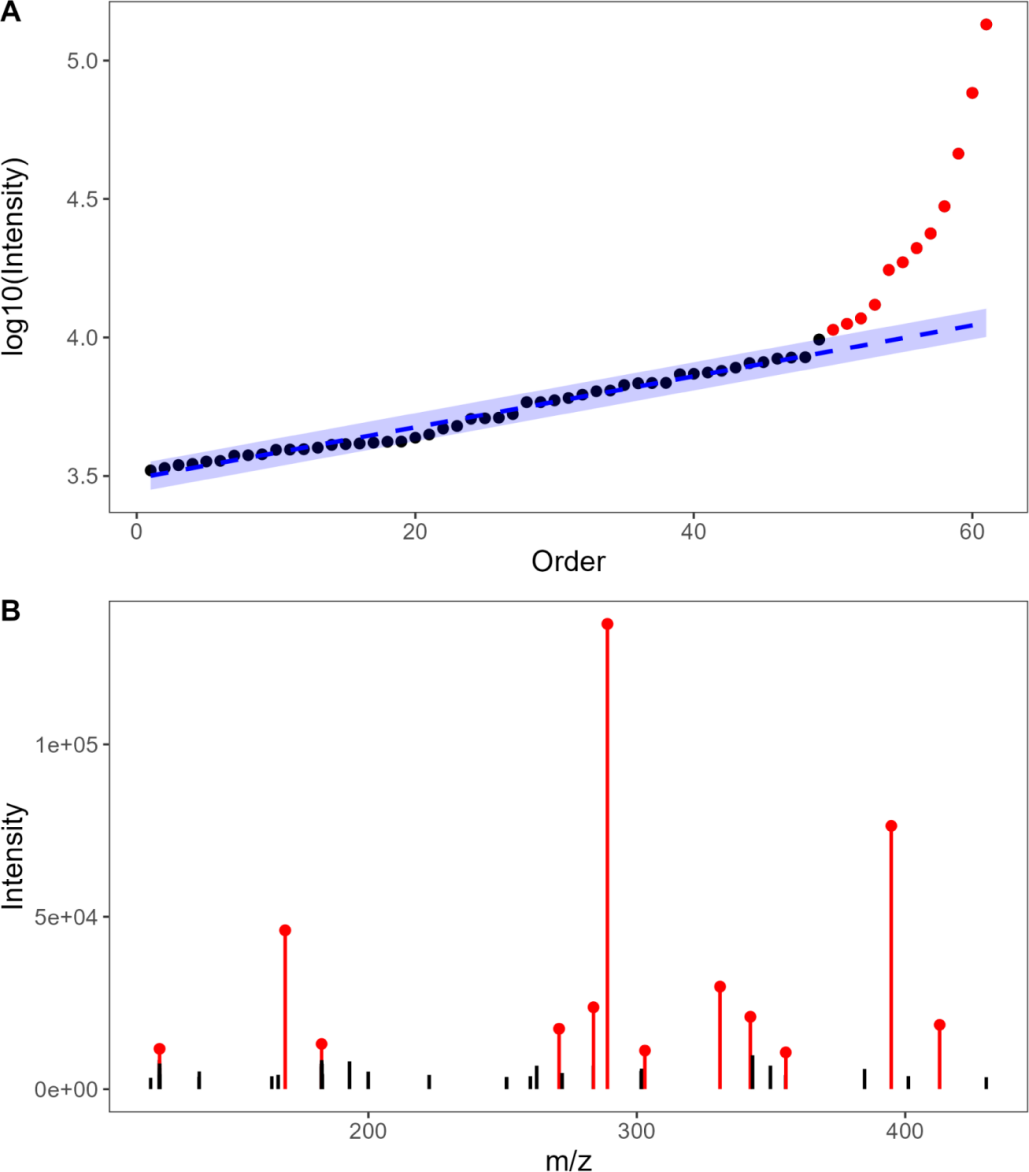
Example of noise cleaning for a catechin-3-*O*-gallate MS/MS spectrum [M – H]^−^ using the tailored noise-cleaning procedure. (A) Rationale behind the proposed noise filter: ions are ordered according to their intensity and a robust linear model is fitted in the first 75th percentile (blue dashed line) where a linear behavior can be observed. Red dots highlight the intensity of the ions kept after the filtering procedure which were outside the 3 SDs boundaries (blue area). (B) Fragmentation spectra of raw data, in red the ions kept after applying the filtering process.

● ions were ordered by increasing intensity, background ions exhibit a linear trend, while true fragment ions deviate from this pattern (Figure 1A);

● a robust linear model (RLM) was fitted for the lowest 75th percentile of intensity-ordered ions;

● the standard deviation (SD) of the RLM residuals was calculated;

● ions with intensity values below the intensity of the last-ranked ion exceeding the upper boundary of the RLM (set at 3 SDs above the trend line) were eliminated as noise, effectively removing the majority of the ions fitting the RLM trend.

R code to apply this approach is available in the Supporting Information (Code S1). If the intensity of all fragment ions fell within the 3 SDs boundaries, the original raw MS/MS spectra were kept unchanged.

### Absolute Cutoff Noise-Cleaning Procedure

For each spectrum, a cutoff threshold was determined by applying a specific percentage relative to the base peak intensity within the spectrum. Ions below this threshold were subsequently filtered using the “filterIntensity” function from the Spectra R package.^20^ To simulate the effect of progressively stricter noise cutoffs, an increasing percentage from 0 to 10% was applied.

### Consensus Spectra Creation

MS/MS spectra of the same compound were selected from data used for in-house library creation. A consensus spectrum was created for each chromatographic run where at least 3 fragmentation spectra of the same compound were present. Ions present in at least 70% of considered MS/MS were retained in the final spectra using “combineSpectra” function from Spectra R package.^20^

### GNPS Score Calculation

Fragmentation spectrum similarity was calculated using the “join-gnps” and “gnps” functions from the R package MsCoreUtils,^20^ with a tolerance mass threshold of 0.01. These functions calculate similarities considering ions that match either for absolute *m*/*z* value or neutral loss.

### Similarity between Homologous MS/MS Spectra

To investigate similarities between different spectra corresponding to the same compound and molecular ion, fragmentation spectra were automatically selected from each acquired file from the injections of analytical standards used for the in-house library creation. This was achieved through peak detection using the XCMS R package.^21^ Chromatographic peaks corresponding to the analytes were identified as the highest intensity peaks matching the analyte *m*/*z* value (only [M + H]^+^ or [M – H]^−^ were considered, with a mass tolerance of 0.01). Fragmentation spectra within a retention time (RT) window defined by the peak’s minimum RT minus 5 s and maximum RT plus 10 s were included. Similarity scores were then calculated for matches between MS/MS spectra of the same compound and ion.

### Molecular Network

Molecular networks (MNs) were constructed for each data set separately: the in-house library, the GNPS Orbitrap data set, the GNPS ToF data set and the biological data set. For each data set, MNs were generated independently for positive and negative ionization modes, based on spectral similarities within each group. For each analyte in the in-house library, a reference MS/MS spectrum for each ionization polarity was manually selected considering [M + H]^+^ and [M – H]^−^ adducts (Table S1) The number of considered spectra for different data sets is listed in Table S2. MNs were visualized by using the VisNetwork R package. To minimize bias from threshold selection, a zero-thresholding approach was applied for edge creation.

### Structural Similarity

To calculate the structural similarity between the molecules, Tanimoto score^22^ was calculated with function “fmcsR” from fmcs R package starting from sdf files created with “smiles2sdf” function of ChemmineR R package.^23^

### Identification of MS/MS Explained Ions

For each data set and ionization polarity, a mascot generic format (MGF) file was created from the spectra object using the function “MsBackendMgf” from the corresponding R package. SIRIUS^24^ software (version 5.8.0) was then used for fragmentation tree computation specifying the known molecular formula and adduct for each compound and applying the default parameters. Ions that could be explained by the SIRIUS fragmentation tree algorithm were considered as potential fragments of the individual compounds.

### Artificial Noise Ions

Artificial MS/MS spectra were generated using the output obtained from SIRIUS processing, considered as “zero-noise spectra”. The filtered spectra using SIRIUS processing were modified by randomly adding low-intensity ions (with values between zero and the value of 5% of the corresponding base ion intensity) in increments of 5 ions, up to a total of 100 added ions. Similarity scores were calculated between the modified spectra with the added noise, using the GNPS score previously indicated.

### Minimum Spanning Tree

A distance matrix was generated by subtracting similarity score values in the MN similarity matrix from value 1. The minimum spanning tree (MST) was constructed using the “mst” function from the iGraph R package.^25^

## Results and Discussion

### Influence of Noise on Similarity Score

In the GNPS similarity score calculation, the overall ion intensity of each spectrum is considered. As noise increases, the relative influence of the most intense peaks is reduced, leading to a lower overall similarity score. To quantify this effect, the impact of a sequential addition of low-intensity ions to “clean” MS/MS spectra of the tripeptide Asp-Gly-Val and of sucrose was investigated. As indicated in the experimental section, “zero-noise” spectra containing only explained peaks were obtained from the processing of the Asp-Gly-Val and sucrose experimental spectra with SIRIUS. The similarity between the “zero-noise” spectra and the ones obtained by adding an increasing number of low-intensity ions is shown in Figure 2A. The figure illustrates a progressive reduction of the similarity score as the number of “noise” ions increases. The decline in similarity score is more pronounced for the tripeptide Asp-Gly-Val compared to sucrose. This difference is likely due to the lower number of explained fragments in the “zero-noise” spectrum of the tripeptide (2 ions) compared to sucrose (12 ions), making it more sensitive to the increase in intensity relative to low-intensity ions.

**Figure 2:**
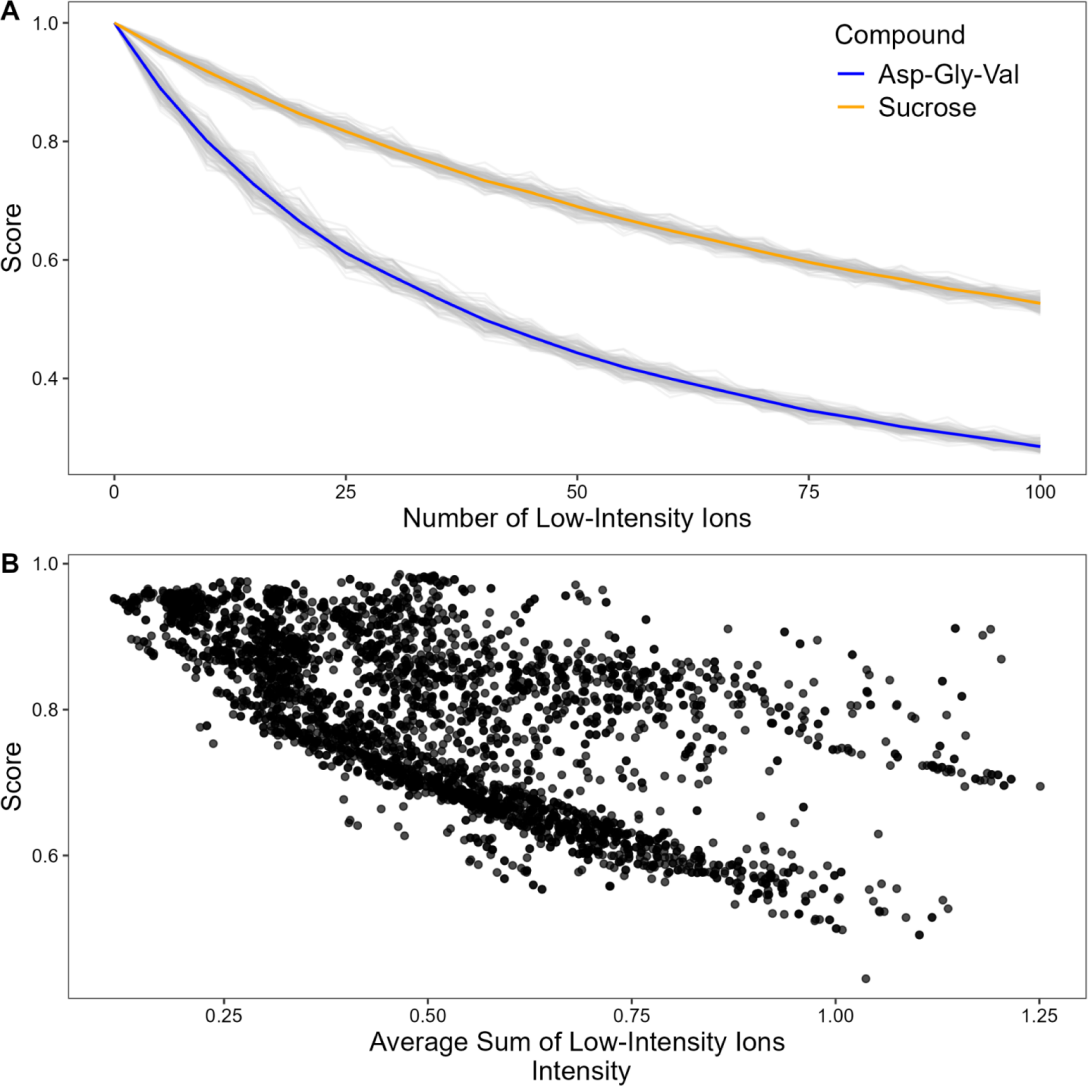
Influence of low-intensity ions on score values. (A) Average similarity score changes as low-intensity ions are artificially added to MS/MS spectra of the tripeptide Asp-Gly-Va (blue line) and sucrose (orange line), based on 100 random noise simulations (gray lines) for each compound. (B) Association between the similarity score of two spectra of the same compound and the average sum of low-intensity ions (considering as low-intensity ions those with an intensity lower than 5% of the base peak intensity; intensity normalized to 1).

This behavior can also be observed in a less artificial context by analyzing the data generated from the injections of all analytical standards. During analysis, the fragmentation spectra of the same compound are indeed typically recorded several times along the chromatographic elution profile. Figure 2B shows how the similarity scores between these spectra relate to the average sum of low-intensity ion signals (those with relative intensities below 5% of the base peak), normalized to the base peak, across all standard injections in the in-house library. A general negative trend can be observed, meaning that the total intensity of low-intensity ions, which are likely to represent noise ions, negatively influences the score similarity value.

### Denoising Effect on Homologous Spectra

Since noise has been shown to strongly influence the similarity score, its elimination is a crucial step in achieving more robust and reliable similarity scores. An example of the effects of noise reduction on the similarity score between two spectra of the same compound is shown in Figure 3. The figure displays a mirror plot comparing two different spectra of procyanidin B3 before and after denoising. Since these spectra belong to the same compound, the similarity score is expected to approach 1. However, the similarity score between the raw spectra reaches only 0.65. After applying the tailored filtering method, the score improves to 0.86. This improvement resulted from the removal of low-intensity ions, which were also those that did not match between the two MS/MS spectra (black colored in Figure 3). As already mentioned, the elimination of nonmatching ions boosted the spectral matching, increasing the similarity score.

**Figure 3:**
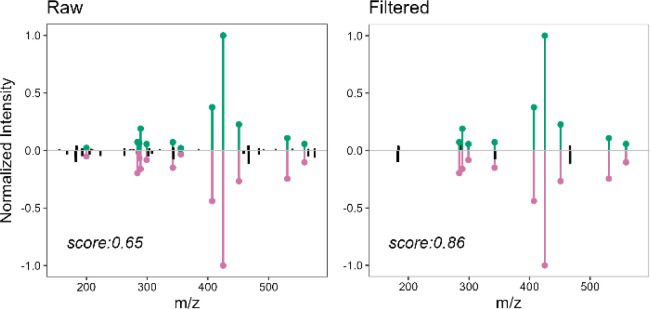
Mirror plot of two different MS/MS spectra of Procyanidin B3 acquired in negative ionization mode, before (left) and after (right) the noise-cleaning procedure. In purple and green matched ions of the first and second spectra, respectively; nonmatched ions are represented in black.

To evaluate the generalized effect of noise elimination on the spectra, similarity scores between the full set of different spectra of the same compound were compared before and after applying different noise-cleaning strategies: (i) the proposed tailored denoising workflow, (ii) a 5% cutoff threshold based on the base ion intensity and (iii) consensus spectrum generation. As mentioned, these comparisons involve spectra of the same compound and ion, so the similarity values are expected to be close to 1. Figure 4 compares the distribution of similarity scores from the matches made before and after the noise filters application. The more evident improvements are relative to the tailored and 5% cutoff denoising approaches, with the majority of matches achieving a score greater than 0.90. Although all filtering methods improve the overall level of similarity, the 5% cutoff method shows a slightly higher median and a narrower data distribution than the other methods. The consensus spectra creation method yields the poorest denoising performance. Moreover, it evaluates fewer matches as, for consensus spectra creation, at least three spectra of the same compound within a chromatographic run are required. That is a condition not always present in the considered data. It is important to highlight that the consensus spectra creation may be challenging in many analytical contexts: multiple fragmentation spectra of the same species may not always be available, particularly for low-intensity precursors. In addition, this approach cannot be applied to public spectral library data, which typically contains only one spectrum per compound.

**Figure 4:**
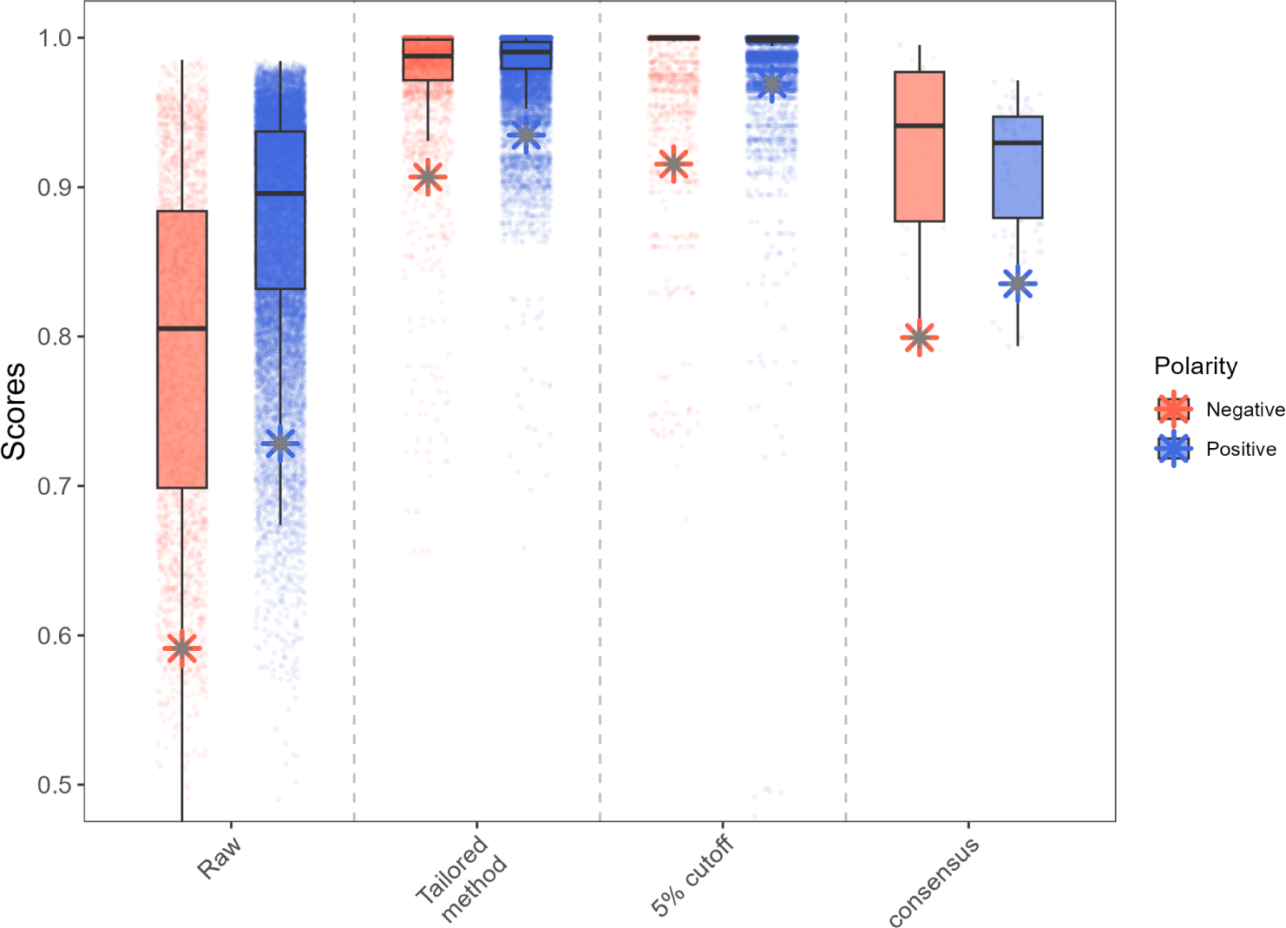
Boxplot showing the distribution of similarity scores for pairwise matches between different MS/MS spectra of the same compound from raw data and after applying three different noise-cleaning methods: (i) tailored denoising workflow, (ii) 5% intensity cutoff and (iii) consensus spectrum generation. The number of evaluated matches is lower for the consensus spectrum approach because its creation requires at least three MS/MS spectra of the same compound, a condition not always met in the data set. The fifth percentile thresholds are indicated with asterisks.

In library comparisons, establishing a threshold for identifying identical spectra is a typical step during the annotation procedure. One way to establish this threshold could be to consider the fifth percentile (asterisks in Figure 4) as the value above which two spectra can be reliably considered the same. The obtained values of raw data are far below the ones observed for denoising methods, especially for the one elaborated with tailored and 5% cutoff denoising methods. These results suggest that noise not only lowers similarity scores but also increases their variability, leading to a broader distribution of scores in raw data.

In MN construction, the threshold also determines whether molecules are connected, through edges, based on their similarity. A common threshold used in MN creation is 0.70.^26^ A non-negligible proportion of raw data scores are below this value, even if in these cases we were comparing spectra from the same compound. This issue is especially problematic for negative ionization data where the fifth percentile of the score of raw data is 0.59. Therefore, even though MN is designed to investigate similar compounds, applying a 0.70 threshold on raw data would also result in the loss of edges between spectra of related compounds, leading to inaccuracies in network construction.

According to this score-wise evaluation, the denoising method based on the 5% cutoff shows the best results. However, it is important to remark that similarity score is primarily determined by the number of matching ions so its value could be increased also removing low-intensity ions that provide valuable structural information. In this case, similarity would be boosted at the price of reducing the information useful for molecular identification and MN reconstruction. The effect of denoising on the loss of possibly informative ions is discussed in the last results section.

### Denoising Effect on Molecular Network

Since noise elimination improves the affordability of results of comparisons between homologous spectra, it is also expected to improve the information content of MN by increasing similarity scores for spectra of structurally similar compounds and decreasing scores for dissimilar ones. Figure 5 illustrates the effect of noise elimination on the GNPS similarity score constructed from the set of our in-house library of analytical standards, before and after applying 5% cutoff noise elimination. As expected, the denoised matrix appears more “contrasted” with well-separated high similarity regions (red or warm colors in Figure 5) superimposed to a more uniform low similarity background (blue or cold colors in Figure 5). What would be indeed expected from a noise removal strategy would be an increase of similarity between compounds with “close” fragmentation patterns with a contemporary decrease in similarity between very different fragmentation patterns.

**Figure 5:**
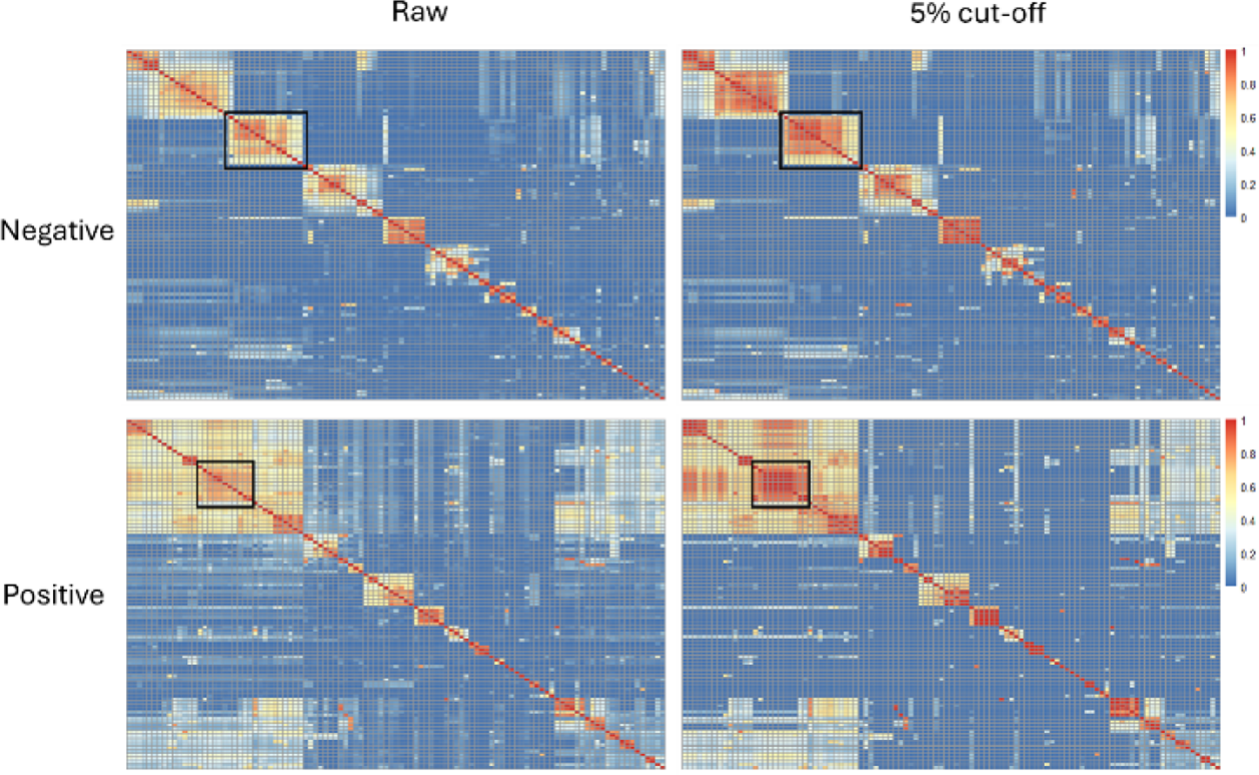
Heatmaps of the score matrices for raw and cleaned data used for MNs creation. Each row and column represents the spectra of one compound and the cells are colored according to the value of the similarity score between them. Cold (from blue) and warm (until red) colors indicate lower and higher similarity scores, respectively. Black rectangles highlights the part with evident changes after applying denoising.

To perform a more quantitative comparison of the matrices, we considered how the noise cleaning process impacted the distribution of the similarity scores included in the two proximity matrices. To do that, the [0,1] interval was cut into 40 bins and we compared the number of entries falling in each bin before and after noise removal. Figure 6 displays the difference in counts in the individual bins between the raw and denoised matrices: bars above zero indicate a higher number of scores in the raw matrix, while bars below zero indicate a higher number in the denoised matrix. The results show the expected increase of the couples of spectra with low similarity values for denoised data. Zero scores are notably more frequent in the cleaned data matrix (e.g., for positive data: 654 zeros in the raw matrix compared to 3,779 in the filtered matrix), suggesting that noise filtering effectively reduces false connections, probably caused by noise-related ions. This leads to a decrease in the similarity score for unrelated spectra, enhancing the overall accuracy of the network. Figure 6 shows that the cleaning process primarily increases scores in the lower range (below 0.025, the first bar in Figure 6), mostly represented by zero values. At the same time, low-value positive scores (between 0.025 and 0.25) are primarily characteristic of raw matrix data. These scores can be considered false positives, as they may result from random matches between noise ions, which do not represent genuine similarities between spectra. Scores above 0.8 are also more frequent in clean data matrices for both ionization modes, indicating stronger similarities among different compounds.

**Figure 6:**
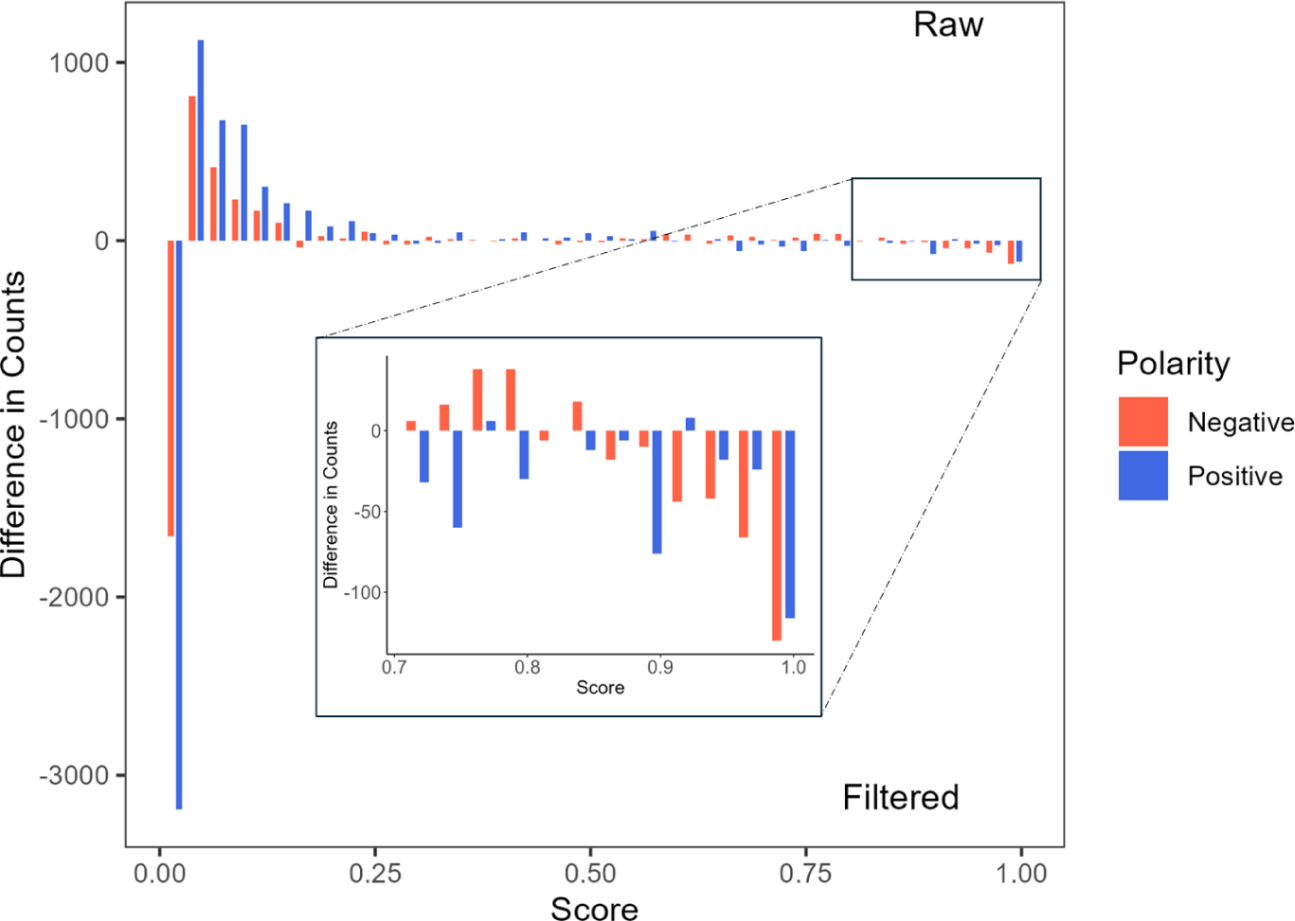
Effect of the noise cleaning on the set of scores of the proximity matrices of Figure 5. Each bar represents a 0.025 bin. Negative values indicate a higher number of occurrences in the denoised matrix.

As a further confirmation of the positive effects of noise elimination, we also calculated the Tanimoto similarity score between the chemical structures of the set of our standards. Remarkably, most zero-value matches in the cleaned data, using the 5% cutoff for noise elimination, are relative to matches between spectra of molecules that also have relatively low Tanimoto similarity values (Figure S1). This suggests that the increase in zero values does not lead to a loss of edges between structurally similar compounds, further confirming that similarities that were nonzero in raw data but became zero in the cleaned data were primarily due to noise random matches in MS/MS spectra. In summary, cleaning reduces edges between dissimilar compounds, reducing false positive edges and resulting in a more reliable and interpretable molecular network.

The previous analysis clearly highlights the impact of the noise filtering on the proximity matrix, but it cannot be easily applied to quantitatively compare different noise removal strategies. In order to overcome such limitations, we suggest a strategy based on the calculation of the minimum spanning tree (MST) of the molecular network constructed from the proximity matrix derived from the similarity matrix. The MST is the subset of edges that connects all the vertices (MS/MS spectra), without any cycles and with the minimum possible total edge weight. In our case, it represents the shortest path that connects all compounds inside the MN. Here we propose to use the distribution of the MST distances as a quantitative representation of the structure of the MN. The effectiveness of the proposed strategy was tested on three data sets of different characteristics: (i) GNPS spectra acquired with ToF instruments, (ii) GNPS spectra acquired with Orbitrap instruments, and (iii) the in-house standard library (Table S2). In all six cases (three data sets with two polarities each one) MST was calculated on the raw spectra and on their denoised counterparts. Denoising was performed both by applying different “fixed” cutoffs (5% and 10% of the base peak intensity) and by applying the proposed tailored method. This was possible because the characteristic trend of the intensity-ordered ion distribution shown in Figure 1 was consistently present across the different instrument types (Figure S2), The MST results for the positive ionization mode of GNPS Tof data set, which is the largest data set considered, are presented in Figure 7, while plots for the other five are included in the Supporting Information (Figure S3). These figures show how denoising affects the trends of the ordered MST distances, The black line in the plot shows the MST distances for the MN using the raw data, while the colored lines show the evolution of the MST when noise removal is applied. The most evident effect of noise removal is the uniform rise of the curves on the left side of the plot, which indicates a larger fraction of high similarity (low MST distances) spectra in the presence of denoising. This larger fraction of low distances is consistent with a more compact network where short distances are “tightened” by the removal of low-intensity ions. Noteworthy, at 5% and 10% denoising levels, the curves flatten toward zero. In the MST, distance of zero (equivalent to similarity of one) indicates a collapse of MN structure, where distinct compounds become indistinguishable. This indicates that strong denoising can lead to overlaps in MN, as observed in the zoomed segment of Figure 7. Limiting the number of zero-distance pairs is essential to preserve meaningful topological information and avoid the collapse of the MN structure. The tailored denoising method (blue line) clearly shows a less extreme effect on the shorter distances, with a limited number of values approaching 0. This continues to suggest a tightening of the network, which, however, is not collapsing. The behavior shown in Figure 7 was consistently observed across all data sets and ionization polarities, as shown in Figure S3. This supports a generalized denoising effect and validates the use of MST as a metric for MN structure evolution across different acquisition instruments and polarities.

**Figure 7:**
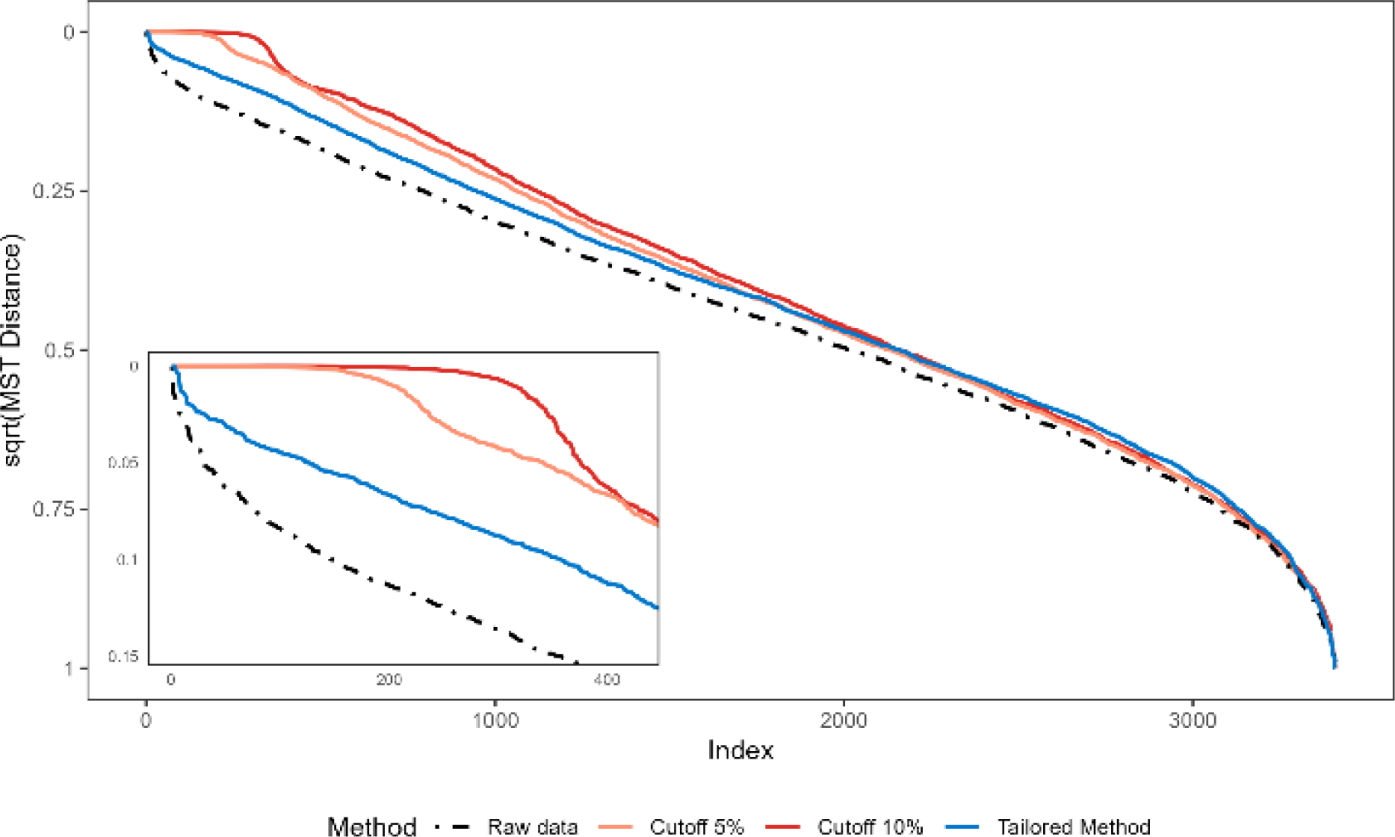
Ordered minimum spanning tree (MST) distances of raw data (black dashed line) compared with MST distances of MNs generated using different percentage cutoffs (red solid lines) and the tailored cleaning method (blue line) for positive ionization modes of GNPS ToF data set. Data are visualized as squared root values to enhance low-magnitude differences in the first part of the plot (i.e., near to 0). On the bottom left, a highlight of the higher MST similarity values.

In order to assess the effect of denoising on the MN structure in a real biological context, we applied MST analysis to MS/MS data from a lipidomic study on vine grape.^18^ MN obtained from the raw spectra were compared to the ones generated after denoising. The resulting ordered MST plots (Figure S4) are in line with what was observed for analytical standards libraries analysis. Denoising resulted in more cohesive MN regions by strengthening connections among lipids within the same class, reflected as increased similarity scores (Table S4). This confirms that denoising enhances spectral similarity between related compounds, thereby improving identification capability. Furthermore, MNs constructed with 5% and 10% cutoff thresholds exhibit longer segments with zero distance values, showing that these denoising methods can also induce a significant collapse of the MN structure when applied to real biological sample data.

### Data-Dependent Denoising Threshold Identification

At the end of the previous section, we demonstrated that intensive noise elimination can make two or more different compounds indistinguishable from their MS/MS spectra. This effect should be clearly limited because it potentially reduces the structural information content of the MN. The point is that strong denoising can also eliminate informative signals from the spectra. The best noise removal strategy should then strike a balance between two contrasting needs: the elimination of signals responsible for false matches and the preservation of potentially informative ions also of relatively low intensity.

In order to investigate these aspects in a quantitative way and to provide a systematic workflow for identifying the optimal noise cutoff SIRIUS software was applied to identify the fragment ions which could be potentially associated with the analyte structure. Even if we cannot claim that these ions are necessarily carrying useful structural information, they are less likely to be part of the noise.

The number of SIRIUS explainable ions was then used as a quantitative proxy of the number of structural ions, while the median of the lowest fifth percentile of the MST distance distribution (the leftmost part in Figure 7) was used as a metric to evaluate MNs structure (*M*(*d*_5%_)). As this value approaches zero, it indicates a collapse of a significant portion (5%) of the network, rendering an important fraction of MS/MS uninformative.

The trends of these two quantities as a function of the noise cutoff (from 0% to 10%) for the positive ionization mode of the GNPS ToF data set are shown in Figure 8. The expected contrasting trends are visible. The rise of the threshold steadily increases the fraction of structurally explained ions which are removed by the noise filter (blue curves, right *y*-axis), conversely the distance among the “closer” compounds reduces, increasing the compactness of the MN (red curves, left *y*-axis). Interestingly, the red curve decreases steeply and already with a threshold of 5% *M*(*d*_5%_) is approaching zero, indicating the collapse of a relevant portion of the MN. As a consequence, also the number of zeros in MST distance, representing the number of indistinguishable MS/MS pairs, is increasing as a function of the severity of the cutoff applied (Figure S5). This analysis shows that the commonly accepted 5% thresholds turned out to be far too aggressive in this case, considering that, over the MN collapse described, it also leads to a loss of almost 50% of the ions which can be explained by the SIRIUS algorithm (Figure 8).

**Figure 8:**
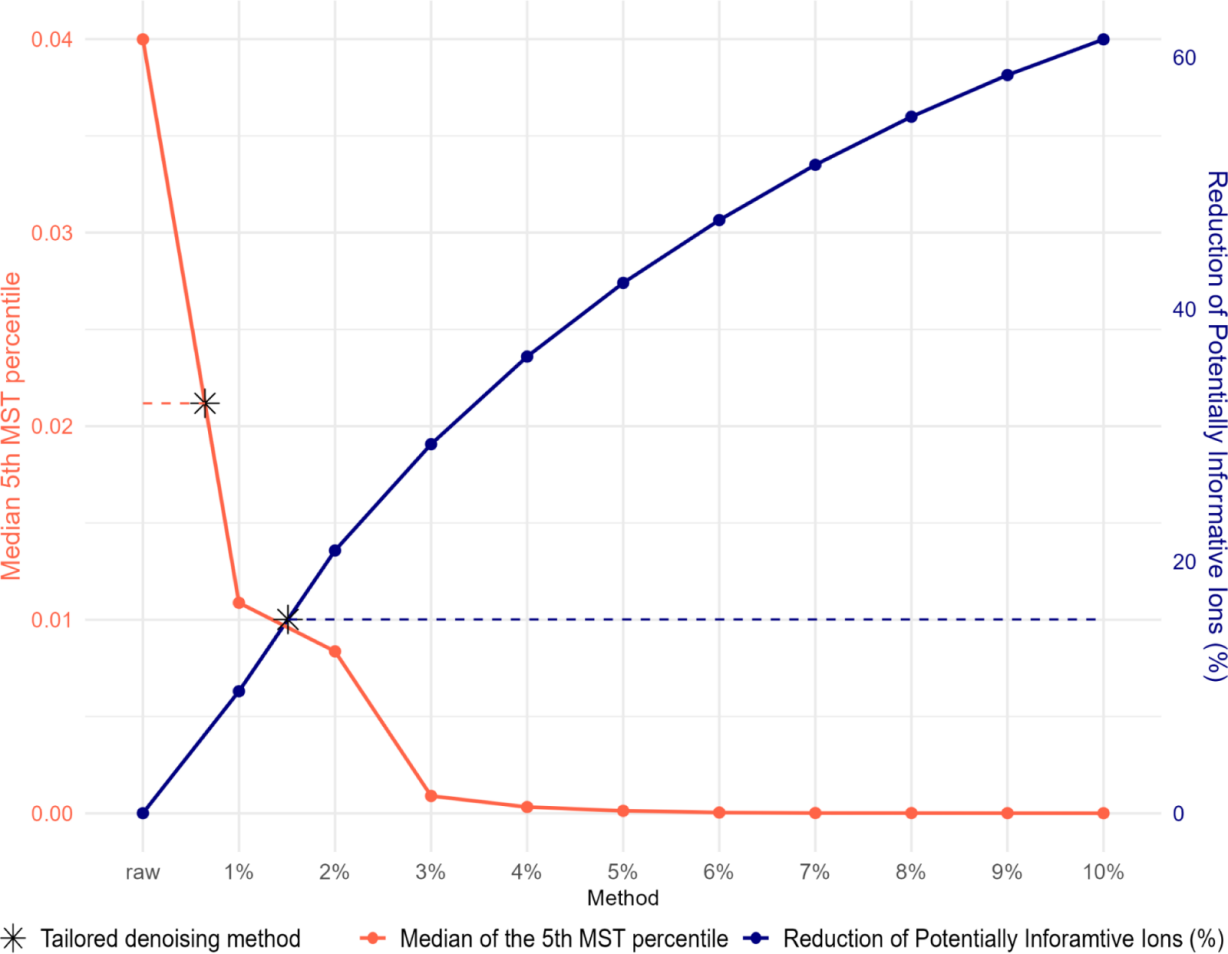
Effect of increasing noise cutoff threshold on the reduction of structurally explained ions and the median of the lowest fifth percentile of the MST distance distribution (*M*(*d*_5%_)) for the positive GNPS ToF data set. The closest cutoff value to the two-line intersection can be taken as an optimized denoising threshold. Results from the tailored data-dependent cleaning procedure are shown in black stars and horizontal dashed lines.

Comparable results are shared across different data sets and polarities analyzed, as shown in Figure S6.

From Figure 8, the ideal noise threshold can be identified as the cutoff closest to the intersection of the two curves. In this specific example, it could be set between 1 and 2%. In the figure, two asterisks highlight the outcomes of the proposed denoising method based on RLM, which showed results comparable to the “optimal” one, in particular as far as the preservation of structurally plausible fragment ions was concerned. In this respect, it is important to highlight that the identification of the optimal noise threshold requires the processing of multiple injections of known compounds, which could not always be feasible. In contrast, the suggested tailored denoising method, based on the creation of a robust linear regression model applied to intensity-ordered MS/MS ions, can be implemented on individual spectra without the need for additional observations or reference materials. Nonetheless, the proposed method is expected to work only if the intensity distribution within a spectrum follows the pattern shown in Figure 1A and this has to be checked for each instrumental setup.

For the majority of analyzed data sets (Figure S6), this approach scored results comparable to those obtained using the method shown in Figure 8, further validating its applicability.

## Conclusion

The impact of noise ions on similarity scores of mass spectra should not be ignored. Noise reduction enhances the affordability of score values for both homologous spectrum comparison and MN. In particular, denoising improves MNs clustering, reducing the number of false-positive connections between nodes relative to chemically dissimilar compounds. Our investigation revealed that MST represents a valid statistical method for a quantitative evaluation of the MN structure. Based on this method, we proposed a workflow to determine data-dependent optimal noise cutoff thresholds producing cleaner molecular networks that retain structurally interpretable fragment ions while preventing collapse of meaningful network portions. The effectiveness of this approach was demonstrated both on an in-house standard library and on two larger open-source MS/MS libraries. Additionally, we introduced a simple, intensity-based denoising method that performs comparably to the optimal threshold in many cases. This approach offers a principled alternative to fixed threshold denoising strategies.

## Supplementary Material



## Notes

Script for the function of the tailored cleaning noise procedure; list of considered compounds used for in-house standards library dataset creation; number of considered MS/MS spectra in network creation and MST evaluation for different datasets; compounds considered for MNs creation from data of a biological study on grape lipids; the top 10 MST connections with the lowest distance (highest spectral similarity) created with the biological dataset of grape lipidomics, processed with the tailored denoising method (PDF)
