# Supplementary material for: Improving Spectral Similarity and Molecular Network Reliability through Noise Signal Filtering in MS/MS Spectra
Source: Anal Chem. 2025 Jul 17;97(29):15873–82. doi: 10.1021/acs.analchem.5c02109 (PMC12311886; doi:10.1021/acs.analchem.5c02109)

# Supplementary Materials

# Improving Spectral Similarity and Molecular Network Reliability through Noise Signal Filtering in MS/MS Spectra.

Nicola Dalla Valle[1,2,3], Mar Garcia Aloy[2], Peter Robatscher[3], Pietro Franceschi[2]* and Michael Oberhuber[3].

[1] University of Trento, 38100 Trento, TN, Italy;

[2] Research Innovation Centre Fondazione Edmund Mach, 38010 San Michele a/A, TN, Italy;

[3] Laimburg Research Centre, Laimburg 6 – Pfatten (Vadena), 39040 Auer (Ora), BZ, Italy;

**Code S1** – Script for the function of the tailored cleaning noise procedure.

```
library(tidyverse)

library(MASS)

library(Spectra)

library(dplyr)


cleaning_ref<- function(ms2){
  ms2_tb <- tibble(
  id = ms2$id,
  dataOrigin = ms2$dataOrigin,
  scanIndex = ms2$scanIndex,
  polarity = ms2$polarity,
  msLevel = ms2$msLevel,
  precursorMz = ms2$precursorMz,
  rtime = ms2$rtime,
  inchi=ms2$inchi,
  precursorIntensity = ms2$precursorIntensity,
  ms2_raw = mapply(cbind, mz(ms2), intensity(ms2), SIMPLIFY = FALSE)
) %>%
  mutate(ms2_raw = map(ms2_raw, function(t){
    colnames(t) <- c("mz", "intensity")
    t %>%
      as.data.frame() %>%
      mutate(mz = as.numeric(mz)) %>%
      mutate(intensity = as.numeric(intensity))
  })) %>%
  mutate(ms2_RLM = map(ms2_raw, function(t){
    t <- t %>%
      filter(intensity>0) %>%
      arrange(intensity) %>%
      mutate(order = seq(nrow(.)))
    p75 <- t %>%
      pull(intensity) %>%
      quantile(0.75)
    u <- t %>%
      filter(intensity < p75)
    if(nrow(u)>3){
```

```
        mdl <- rlm(data = u, log10(intensity) ~ order)
    t_filt<- t %>%
      mutate(pred = predict(mdl, t)) %>%
      mutate(res = abs(pred - log10(intensity))) %>%
      filter(res > sd(residuals(mdl))*3) %>%
      arrange(mz)
    if (nrow(t_filt) > 0) {
      t<- t_filt
    } else {
     t<- t %>% arrange(mz)
    }
  } else {
    return(t %>% arrange(mz))  # Not enough points to model
  }
  }))
ms2_cl <- ms2_tb %>%
  mutate(precursorIntensity = precursorIntensity) %>%
  mutate(
    mz = map(ms2_RLM, ~ .x$mz),
    intensity = map(ms2_RLM, ~ .x$intensity)
  ) %>%
  dplyr::select(
    id, dataOrigin, scanIndex, inchi, rtime, precursorMz, precursorIntensity,
    polarity, mz, intensity
  ) %>%
    filter(map_lgl(mz, ~ length(.x) > 0)) %>%
  as.data.frame() %>%
  Spectra()
return(ms2_cl)
}
```

**Table S1**- List of considered compounds used for in-house standards library dataset creation, with their presence in positive and negative ionization data, and the number relative to the chromatographic method used in data acquisition. “I”,”II” and “III” are relative to the different compound isomers.

method 1 - Kinetex C18 (150 mm x 2.1 mm I.D.), particle size 2.6 µm, Phenomenex Torrance, CA, USA); phase A: water with 0.1% formic acid; phase B: acetonitrile with 0.1% formic acid; gradients: 0–1 min 5% B; 1–7 min linear gradient 5–45 % B; 7–8.5 min linear gradient 45–80 % B; 8.5–10.5 min mantaining 80% B; 10.5–11 min linear gradient from 80% B to 5% B; 11–12 min maintaining 5% B.

method 2 -Kinetex C18 (150 mm x 2.1 mm I.D.), particle size 2.6 µm, Phenomenex Torrance, CA, USA); phase A: water with 0.1% formic acid; phase B: acetonitrile with 0.1% formic acid; gradients: 0–1 min 5% B; 1–12.5 min linear gradient from 5% to 100% B; 12.5–14 min maintaining 100% B; 14–14.3 min linear gradient 100–5 % B; 14.3–15.3 min 5% B.

method 3  - column: ACQUITY Premier (HSS T 3.1 µm VanGuard FIT, 2.1 x 100 mm); phase A: milliQ water + 0.1% formic acid; phase B: acetonitrile + 0.1% FA; gradients: B at 0%; B at 5% in 1 minute, then reaching 45% in 7 minutes. 100% B from minute 7 to minute 9.3 kept at 100% to minute 11, and re-equilibration at 0% B for 3 additional minutes.

method 4 - column: acquity UPLC CSH-C18 (2.1 × 100 mm, 1.7 µm); pahse A:1 mM ammonium acetate in 5:95 water/acetonitrile (v/v), phase B: 1 mM ammonium acetate in 50:50 water/acetonitrile (v/v); gradients: 0 min 0.1% B; 0–6 min 6% B; 6–10 min increase to 25% B; 10–11 min 98% B; 11–13 min 100% B, held until 18.6 min. Then at 18.6–18.7 min the percentage of B decreased to 0.1% and remained constant until 24 min.

| compound | chromatographic method | positive ionization | negative ionization |
|---|---|---|---|
| 2,4-Dihydroxybenzoic acid | 1,2 | - | X |
| 2,5-Dihydroxybenzoic acid | 1,2 | - | X |
| 2-Furoylglycine | 1,2 | X | - |
| 3,4-Dihydroxy-phenyl-valerolactone | 1,2 | X | X |
| 3,4-Dihydroxybenzoic acid | 1,2,3 | - | X |

| | | | |
|---|---|---|---|
| 3,4-Dihydroxycinnamic Acid (Caffeic Acid) | 1 | - | X |
| 3,4-Dihydroxyphenylglycol | 1 | - | X |
| 3,5-Dihydroxycinnamic acid | 1 | - | X |
| 5-(3',5'-dihydroxyphenyl)-y-valerolactone | 1,2 | X | - |
| 5-(3',5'-dihydroxyphenyl)-y-valerolactone-3'-O-glucuronide | 1,2 | X | X |
| 5-(3',5'-dihydroxyphenyl)-y-valerolactone-3',4'-O-sulphate (I) | 1,2 | - | X |
| 5-(3'-hydroxyphenyl)-y-valerolactone-3'-O-glucuronide | 1,2 | X | - |
| 5-(3',4'-hydroxyphenyl)-y-valerolactone (I) | 1,2 | X | - |
| 5-(3',4'-hydroxyphenyl)-y-valerolactone (II) | 1,2 | X | - |
| 5-(3',4'-hydroxyphenyl)-y-valerolactone-3',4'-O-sulphate (I) | 1,2 | X | X |
| 5-(3',4'-hydroxyphenyl)-y-valerolactone-3',4'-O-sulphate (II) | 1,2 | X | X |
| 5-Tertbutyl-methyl-3-furoic acid | 1,2 | X | - |
| Acetylglycine | 1,2 | X | - |
| Acetylornithine | 1,2 | X | X |
| Ala-Asp | 1,2 | X | - |
| Ala-Leu | 1,2 | X | - |
| Ala-Phe | 1,2 | X | - |
| Ala-Pro | 1,2 | X | - |
| Ala-Tyr | 1,2 | X | - |
| Amyl-2-furoate | 1,2 | X | - |
| Apigenin 7,4'-dimethyl ether | 3 | X | - |
| Apigenin-7-O glucoside | 3 | X | X |
| Ascorbic acid | 1,2 | X | X |
| Asn-Asp-Val | 1,2 | X | X |
| Asp-Asn-Val | 1,2 | X | X |
| Asp-Asp-Val | 1,2 | X | X |
| Asp-Gly | 1,2 | X | - |
| Asp-Gly-Ile | 1,2 | X | - |
| Asp-Gly-Leu | 1,2 | X | X |
| Asp-Gly-Val | 1,2 | X | X |
| Asp-Ile | 1,2 | X | X |
| Asp-Leu | 1,2 | X | X |
| Asp-Met | 1,2 | X | X |
| Asp-Phe | 1,2 | X | - |
| Asp-Tyr | 1,2 | X | X |
| Asp-Val | 1,2 | X | X |
| Aspartic acid | 1,2 | X | - |
| Butyric acid ethyl-ester | 1,2 | X | - |
| Carnosic acid | 3 | - | X |
| Carnosol | 3 | X | X |
| Carvacrol | 1,2 | X | - |
| Cer d18:0/16:0 | 4 | - | X |
| Cer d18:1/16:0 | 4 | - | X |
| Chrysoeriol | 1,3 | X | X |
| Circimaritin | 3 | X | X |
| Diosmetin | 1 | X | X |
| Dodecanedioic acid | 1,2 | X | X |
| Dopamine | 1,2 | X | - |
| Dulcitol | 1 | - | X |
| Epicatechin-3-O-gallate | 1 | - | X |
| Epigallocatechine gallate | 1 | - | X |
| Eupatorin-5-methyl-ether | 3 | X | X |
| Fertaric acid | 1,3 | - | X |
| Folinic acid | 1,2 | X | X |
| Fructose | 1 | - | X |
| Galactinol | 1 | - | X |
| Gallic acid | 1 | X | - |
| Genkwanin | 3 | X | X |
| Glucose | 1 | - | X |
| Glutamine | 1,2 | X | - |
| Gly-Glu | 1,2 | X | - |
| Gly-HVal | 1,2 | X | X |
| Gly-Val | 1,2 | X | X |
| Gly-Val-Gly | 1,2 | X | X |
| Guanosine 5'-monophosphate disodium salt | 1 | - | X |
| HPro-Pro | 1,2 | X | - |
| HVal-Gly | 1,2 | X | X |
| Hesperidin | 3 | - | X |

| | | | |
|---|---|---|---|
| HexCer d18:1/18:0 | 4 | - | X |
| Hispidulin | 3 | X | X |
| Hydroxytyrosol | 1 | - | X |
| Hypaphorine | 2 | X | - |
| Hyperoside / Quercetin 3-O-galactoside | 1 | - | X |
| Ile-Pro-Ile | 1 | X | X |
| Isoferulic acid | 1,2 | X | - |
| Isoleucine | 1,2 | X | - |
| Isorhamnetin | 1 | X | - |
| Isorhamnetin-3-O-glucoside | 3 | X | X |
| Isorhamnetin-3-rutinoside | 1 | - | X |
| Kaempferol-3-Glucoside | 1,3 | X | X |
| Kaempferol-3-O-rutinoside | 3 | X | X |
| L-3,4-Dihydroxyphenylalanine methyl esther hydrochloride | 1 | X | - |
| L-Pipecolic acid | 1 | X | - |
| LPC(16:0) | 4 | X | - |
| LPC(18:0) | 4 | X | - |
| LPC(18:1) | 4 | X | - |
| LPC(18:2) | 4 | X | - |
| LPC(18:3) | 4 | X | - |
| Lactose | 1 | - | X |
| Leu-Ala | 1,2 | X | - |
| Leu-Leu-Leu | 1 | X | - |
| Leu-Ser-Phe | 1,2 | X | X |
| Leucine | 1,2 | X | - |
| Loganin | 1 | X | - |
| Lysine | 1,2 | X | - |
| Malic acid | 1 | - | X |
| Mannitol | 1 | - | X |
| Mannose | 1 | - | X |
| Met-Pro | 1 | X | - |
| Methyl-pelargonate | 1,2 | X | - |
| Naringenin-4',7-dimethyl ether | 3 | X | - |
| Naringenin-7-O-glucoside | 3 | X | - |
| Oleuropein | 1 | - | X |
| Ophtalmic acid | 1,2 | X | - |
| PA(16:0/18:2) | 4 | - | X |
| PE(16:0/18:2) | 4 | - | X |
| PE(16:0/18:3) | 4 | - | X |
| PE(18:1/18:1) | 4 | - | X |
| PE(18:1/18:2) | 4 | X | X |
| PE(18:1/18:3) / PE(18:2/18:2) | 4 | X | - |
| PE(18:2/18:3) | 4 | X | X |
| PG(16:0/18:3) | 4 | - | X |
| PG(18:1/18:2) | 4 | - | X |
| PI(18:0/18:2) | 4 | - | X |
| PI(18:2/18:2) | 4 | - | X |
| PS(18:1/18:3) / PS(18:2/18:2) | 4 | X | - |
| Phe-Ala | 1,2 | X | - |
| Phe-Ser-Phe | 1,2 | X | X |
| Phe-Thr | 1,2 | X | X |
| Phe-Thr-Phe | 1,2 | X | X |
| Phenylalanine | 1,2 | X | - |
| Phlorizin | 3 | - | X |
| Procyanidin B1 | 1 | - | X |
| Procyanidin B2 | 1 | - | X |
| Procyanidin B3 | 1 | - | X |
| Quercetin-3-O-glucopyranoside | 1 | - | X |
| Quercetin-3-glucoside | 3 | X | X |
| Quercitrin /Quercetin-3-rhamnoside | 1 | - | X |
| Raffinose | 1 | - | X |
| Rosmarinic acid | 3 | - | X |
| S-Methyl-L-cysteine | 1 | X | - |
| SM(d18:1/18:0) | 4 | X | - |
| Salvigenin | 3 | X | - |
| Sorbifolin | 3 | X | X |
| Sorbitol | 1 | - | X |
| Stachyose | 1 | - | X |
| Succinic acid | 1 | - | X |
| Sucrose | 1 | - | X |

| | | | |
|---|---|---|---|
| Syringic acid | 1,3 | - | X |
| Thr-Phe | 1,2 | X | - |
| Thymol | 1,2 | X | - |
| Tyr-Ala | 1,2 | X | - |
| Tyr-Phe | 1 | X | X |
| Val-Gly | 1,2 | X | - |
| Val-Gly-Gly | 1,2 | X | - |
| Valine | 1,2 | X | - |
| Verbascoside | 1 | - | X |
| a-Hydroxyhippuric acid | 1 | - | X |
| alpha-CEHC | 1,2 | X | X |
| delta-CEHC | 1,2 | X | X |
| gamma-CEHC | 1,2 | X | X |
| o-Hydroxyhippuric acid | 1 | X | X |
| p-Coumaric acid | 1,3 | X | X |
| p-Hydroxyhippuric acid | 1 | - | X |
| trans-Ferulic acid | 1,2,3 | X | - |

**Table S2 -** Number of considered MS/MS spectra in network creation and MST evaluation for different datasets.

| Ionization Polarity | In-house library | GNPS – ToF | GNPS - Orbitrap | Biological dataset |
|---|---|---|---|---|
| Positive | 107 | 3409 | 806 | 208 |
| Negative | 101 | 824 | 275 | 141 |

**Table S3 –** Compounds considered for MNs creation from data of a biological study on grape lipids. For each sample a spectra from raw data was selected according to m/z and retention time values.

| Name | Formula | RT (min) | NEG_adduct | POS_adduct |
|---|---|---|---|---|
| LPC 16:0 | C24H50NO7P | 8.07 | [M+CHO2]- | [M+H]+ |
| LPC 18:0 | C26H54NO7P | 10.23 | [M+CHO2]- | [M+H]+ |
| LPC 18:1 | C26H52NO7P | 8.34 | [M+CHO2]- | [M+H]+ |
| LPC 18:2 | C26H50NO7P | 7.05 | [M+CHO2]- | [M+H]+ |
| LPC 18:3 | C26H48NO7P | 5.55 | [M+CHO2]- | - |
| LPE 16:0 | C21H44NO7P | 8.17 | [M-H]- | [M+H]+ |
| LPE 18:2 | C23H44NO7P | 7.14 | [M-H]- | [M+H]+ |
| PA 16:0_16:1 | C35H67O8P | 16.12 | [M-H]- | [M+NH4]+ |
| PA 14:0_18:2 | C35H65O8P | 15.39 | [M-H]- | [M+NH4]+ |
| PA 15:1_18:2 / PA 15:0_18:3 | C36H65O8P | 15.34 | [M-H]- | - |
| PA 16:0_18:1 | C37H71O8P | 16.9 | [M-H]- | [M+NH4]+ |
| PA 16:0_18:2 | C37H69O8P | 16.23 | [M-H]- | [M+NH4]+ |
| PA 16:0_18:3 | C37H67O8P | 15.66 | [M-H]- | [M+NH4]+ |
| PA 18:0_18:1 | C39H75O8P | 17.71 | [M-H]- | - |
| PA 18:1_18:1 | C39H73O8P | 17.13 | [M-H]- | [M+NH4]+ |
| PA 18:0_18:2 | C39H73O8P | 17.36 | [M-H]- | - |
| PA 18:1_18:2 | C39H71O8P | 16.36 | [M-H]- | [M+NH4]+ |
| PA 18:0_18:3 | C39H71O8P | 16.57 | [M-H]- | - |
| PA 18:2_18:2 | C39H69O8P | 15.7 | [M-H]- | [M+NH4]+ |
| PA 18:2_18:3 | C39H67O8P | 15.13 | [M-H]- | [M+NH4]+ |
| PA 18:3_18:3 | C39H65O8P | 14.57 | [M-H]- | [M+NH4]+ |
| PA 18:2_20:0 | C41H77O8P | 17.97 | [M-H]- | - |
| mPA 16:0_18:1 | C38H73O8P | 16.83 | [M-H]- | [M+NH4]+ |
| mPA 16:0_18:2 | C38H71O8P | 16.23 | [M-H]- | [M+NH4]+ |
| mPA 16:0_18:3 | C38H69O8P | 15.68 | [M-H]- | [M+NH4]+ |
| mPA 18:0_18:1 | C40H77O8P | 17.55 | [M-H]- | - |
| mPA 18:1_18:1 | C40H75O8P | 16.91 | - | [M+NH4]+ |
| mPA 18:1_18:1 / mPA 18:0_18:2 | C40H75O8P | 17.02 | [M-H]- | [M+NH4]+ |
| mPA 18:1_18:2 | C40H73O8P | 16.34 | [M-H]- | [M+NH4]+ |
| mPA 18:2_18:2 | C40H71O8P | 15.72 | [M-H]- | [M+NH4]+ |

| | | | | |
|---|---|---|---|---|
| mPA 18:2_18:3 | C40H69O8P | 15.15 | [M-H]- | [M+NH4]+ |
| mPA 18:3_18:3 | C40H67O8P | 14.55 | [M-H]- | [M+NH4]+ |
| mPA 18:2_20:0 | C42H79O8P | 17.73 | [M-H]- | - |
| mPA 18:2_22:0 | C44H83O8P | 18.38 | [M-H]- | [M+NH4]+ |
| mPA 18:2_23:0 | C45H85O8P | 18.65 | [M-H]- | - |
| PC 30:0 | C38H76NO8P | 16.11 | - | [M+H]+ |
| PC 31:3 | C39H72NO8P | 14.65 | [M+CHO2]- | - |
| PC 16:0_16:0 | C40H80NO8P | 16.97 | [M+CHO2]- | [M+H]+ |
| PC 16:0_16:1 | C40H78NO8P | 16.3 | - | [M+H]+ |
| PC 14:0_18:2 | C40H76NO8P | 15.59 | [M+CHO2]- | [M+H]+ |
| PC 32:3 | C40H74NO8P | 15 | - | [M+H]+ |
| PC 33:2 | C41H78NO8P | 16.1 | - | [M+H]+ |
| PC 33:3 | C41H76NO8P | 15.48 | - | [M+H]+ |
| PC 33:4 | C41H74NO8P | 14.61 | - | [M+H]+ |
| PC 33:5 | C41H72NO8P | 14.21 | [M+CHO2]- | - |
| PC 34:0 | C42H84NO8P | 17.74 | - | [M+H]+ |
| PC 16:0_18:1 | C42H82NO8P | 17.08 | [M+CHO2]- | [M+H]+ |
| PC 16:0_18:2 | C42H80NO8P | 16.49 | [M+CHO2]- | [M+H]+ |
| PC 16:0_18:3 | C42H78NO8P | 15.86 | [M+CHO2]- | [M+H]+ |
| PC 34:4 | C42H76NO8P | 15.16 | - | [M+H]+ |
| PC 35:1 | C43H84NO8P | 17.49 | - | [M+H]+ |
| PC 17:0_18:2 | C43H82NO8P | 16.9 | [M+CHO2]- | [M+H]+ |
| PC 35:3 | C43H80NO8P | 16.42 | - | [M+H]+ |
| PC 35:4 | C43H78NO8P | 15.62 | - | [M+H]+ |
| PC 35:5 | C43H76NO8P | 14.98 | - | [M+H]+ |
| PC 18:0_18:1 | C44H86NO8P | 17.81 | [M+CHO2]- | [M+H]+ |
| PC 18:1_18:1 | C44H84NO8P | 17.17 | - | [M+H]+ |
| PC 18:0_18:2 | C44H84NO8P | 17.28 | [M+CHO2]- | [M+H]+ |
| PC 18:1_18:2 | C44H82NO8P | 16.59 | [M+CHO2]- | [M+H]+ |
| PC 18:0_18:3 | C44H82NO8P | 16.79 | [M+CHO2]- | [M+H]+ |
| PC 18:2_18:2 | C44H80NO8P | 15.97 | [M+CHO2]- | [M+H]+ |
| PC 18:2_18:3 | C44H78NO8P | 15.37 | [M+CHO2]- | [M+H]+ |
| PC 18:3_18:3 | C44H76NO8P | 14.73 | [M+CHO2]- | [M+H]+ |
| PC 18:2_20:0 | C46H88NO8P | 18 | [M+CHO2]- | [M+H]+ |
| PC 38:3 | C46H86NO8P | 17.35 | - | [M+H]+ |
| PE 32:2 | C37H70NO8P | 15.88 | - | [M+H]+ |
| PE 16:0_18:1 | C39H76NO8P | 17.3 | [M-H]- | [M+H]+ |
| PE 16:0_18:2 | C39H74NO8P | 16.72 | [M-H]- | [M+H]+ |
| PE 16:0_18:3 | C39H72NO8P | 16.16 | [M-H]- | [M+H]+ |
| PE 18:0_18:1 | C41H80NO8P | 17.99 | [M-H]- | - |
| PE 18:0_18:2 | C41H78NO8P | 17.49 | [M-H]- | [M+H]+ |
| PE 18:1_18:2 | C41H76NO8P | 16.81 | [M-H]- | [M+H]+ |
| PE 18:0_18:3 | C41H76NO8P | 17 | [M-H]- | [M+H]+ |
| PE 18:2_18:2 | C41H74NO8P | 16.2 | [M-H]- | [M+H]+ |
| PE 18:2_18:3 | C41H72NO8P | 15.61 | [M-H]- | [M+H]+ |
| PE 18:3_18:3 | C41H70NO8P | 14.99 | [M-H]- | [M+H]+ |
| PE 18:2_20:0 | C43H82NO8P | 18.19 | [M-H]- | [M+H]+ |
| PE 18:3_20:0 | C43H80NO8P | 17.72 | [M-H]- | - |
| PE 18:2_21:0 | C44H84NO8P | 18.5 | [M-H]- | [M+H]+ |
| PE 18:2_22:0 | C45H86NO8P | 18.81 | [M-H]- | [M+H]+ |
| PE 18:3_22:0 | C45H84NO8P | 18.41 | [M-H]- | [M+H]+ |
| PE 18:2_23:0 | C46H88NO8P | 19.09 | [M-H]- | [M+H]+ |
| PE 18:2_24:0 | C47H90NO8P | 19.36 | [M-H]- | [M+H]+ |
| PE 43:2 | C48H92NO8P | 19.63 | - | [M+H]+ |
| PG 16:0_16:0 | C38H75O10P | 16.33 | [M-H]- | [M+NH4]+ |
| PG 16:0_16:1 | C38H73O10P | 16.06 | [M-H]- | - |
| PG 16:0_18:0 | C40H79O10P | 17.11 | [M-H]- | - |
| PG 16:0_18:1 | C40H77O10P | 16.43 | [M-H]- | [M+NH4]+ |

| | | | | |
|---|---|---|---|---|
| PG 16:0_18:2 | C40H75O10P | 15.85 | [M-H]- | [M+NH4]+ |
| PG 16:0_18:3 | C40H73O10P | 15.28 | [M-H]- | [M+NH4]+ |
| PI 29:2 | C38H69O13P | 13.61 | [M-H]- | - |
| PI 16:0_16:0 | C41H79O13P | 16.09 | [M-H]- | - |
| PI 32:2 | C41H75O13P | 14.82 | [M-H]- | - |
| PI 16:0_18:1 | C43H81O13P | 16.21 | [M-H]- | - |
| PI 16:0_18:2 | C43H79O13P | 15.61 | [M-H]- | [M+NH4]+ |
| PI 16:0_18:3 | C43H77O13P | 14.99 | [M-H]- | [M+NH4]+ |
| PI 18:0_18:2 | C45H83O13P | 16.46 | [M-H]- | - |
| PI 18:2_18:2 | C45H79O13P | 15.07 | [M-H]- | [M+NH4]+ |
| PI 18:2_18:3 | C45H77O13P | 14.46 | [M-H]- | [M+NH4]+ |
| PI 18:3_18:3 | C45H75O13P | 13.8 | [M-H]- | - |
| PS 34:0 | C40H78NO10P | 16.16 | - | [M+H]+ |
| PS 18:2_20:0 | C44H82NO10P | 17.3 | [M-H]- | - |
| PS 18:2_21:0 | C45H84NO10P | 17.67 | [M-H]- | [M+H]+ |
| PS 18:2_22:0 | C46H86NO10P | 17.99 | [M-H]- | [M+H]+ |
| PS 18:3_22:0 | C46H84NO10P | 17.56 | [M-H]- | - |
| PS 18:2_23:0 | C47H88NO10P | 18.29 | [M-H]- | - |
| Cer;O3 40:1 | C40H79NO4 | 18.65 | [M-H]- | - |
| Cer;O3 41:1 | C41H81NO4 | 18.97 | [M-H]- | - |
| Cer;O3 42:1 | C42H83NO4 | 19.26 | [M-H]- | - |
| Cer;O4 18:0_22:0 | C40H81NO5 | 18.7 | [M-H]- | - |
| Cer;O4 18:1_22:0 | C40H79NO5 | 18.39 | [M-H]- | [M+H]+ |
| Cer;O4 41:0 | C41H83NO5 | 18.98 | [M-H]- | - |
| Cer;O4 18:1_23:0 | C41H81NO5 | 18.72 | [M-H]- | - |
| Cer;O4 18:0_24:0 | C42H85NO5 | 19.27 | [M-H]- | - |
| Cer;O4 18:1_24:0 | C42H83NO5 | 19.02 | [M-H]- | [M+H]+ |
| Cer;O4 44:1 | C44H87NO5 | 19.59 | [M-H]- | [M+H]+ |
| HexCer;O3 33:2 | C39H73NO9 | 15.19 | [M+CHO2]- | [M+H]+ |
| HexCer;O3 34:1 | C40H77NO9 | 16.01 | [M+CHO2]- | [M+H]+ |
| HexCer;O3 34:2 | C40H75NO9 | 15.69 | [M+CHO2]- | - |
| HexCer;O3 34:3 | C40H73NO9 | 14.96 | - | [M+H]+ |
| HexCer;O3 38:2 | C44H83NO9 | 17.47 | - | [M+H]+ |
| HexCer;O3 40:2 | C46H87NO9 | 18.15 | [M+CHO2]- | [M+H]+ |
| HexCer;O3 41:2 | C47H89NO9 | 18.48 | [M+CHO2]- | [M+H]+ |
| HexCer;O3 42:2 | C48H91NO9 | 18.79 | [M+CHO2]- | [M+H]+ |
| HexCer;O3 43:2 | C49H93NO9 | 19.06 | [M+CHO2]- | - |
| HexCer;O3 44:2 | C50H95NO9 | 19.35 | [M+CHO2]- | [M+H]+ |
| HexCer;O4 39:1 | C45H87NO10 | 17.55 | [M+CHO2]- | - |
| HexCer;O4 40:1 | C46H89NO10 | 17.91 | [M+CHO2]- | [M+H]+ |
| HexCer;O4 41:1 | C47H91NO10 | 18.24 | [M+CHO2]- | [M+H]+ |
| HexCer;O4 42:1 | C48H93NO10 | 18.56 | [M+CHO2]- | [M+H]+ |
| HexCer;O4 43:1 | C49H95NO10 | 18.86 | [M+CHO2]- | [M+H]+ |
| HexCer;O4 44:1 | C50H97NO10 | 19.14 | [M+CHO2]- | [M+H]+ |
| HexCer;O4 45:1 | C51H99NO10 | 19.44 | - | [M+H]+ |
| DGMG 18:2 | C33H58O14 | 6.49 | [M+CHO2]- | [M+NH4]+ |
| DGMG 18:3 | C33H56O14 | 5.05 | [M+CHO2]- | [M+NH4]+ |
| MGDG 16:0_18:1 | C43H80O10 | 17.67 | [M+CHO2]- | - |
| MGDG 36:0 | C45H86O10 | 18.9 | - | [M+NH4]+ |
| MGDG 18:1_18:1 | C45H82O10 | 17.75 | [M+CHO2]- | [M+NH4]+ |
| MGDG 18:1_18:2 | C45H80O10 | 17.2 | [M+CHO2]- | [M+NH4]+ |
| MGDG 18:0_18:3 | C45H80O10 | 17.38 | [M+CHO2]- | - |
| MGDG 18:2_18:2 | C45H78O10 | 16.62 | [M+CHO2]- | [M+NH4]+ |
| MGDG 18:2_18:3 | C45H76O10 | 16.05 | [M+CHO2]- | [M+NH4]+ |
| MGDG 18:3_18:3 | C45H74O10 | 15.45 | [M+CHO2]- | [M+NH4]+ |
| DGDG 34:0 | C49H92O15 | 17.73 | [M+CHO2]- | [M+NH4]+ |
| DGDG 34:2 | C49H88O15 | 16.51 | [M+CHO2]- | - |
| DGDG 16:0_18:3 | C49H86O15 | 15.94 | [M+CHO2]- | - |

| | | | | |
|---|---|---|---|---|
| DGDG 18:1_18:2 | C51H90O15 | 16.61 | [M+CHO2]- | - |
| DGDG 18:0_18:3 | C51H90O15 | 16.8 | [M+CHO2]- | [M+NH4]+ |
| DGDG 18:2_18:2 | C51H88O15 | 16 | [M+CHO2]- | [M+NH4]+ |
| DGDG 18:2_18:3 | C51H86O15 | 15.4 | [M+CHO2]- | [M+NH4]+ |
| DGDG 18:3_18:3 | C51H84O15 | 14.77 | [M+CHO2]- | [M+NH4]+ |
| SQDG 16:0_18:1 | C43H80O12S | 16.13 | - | [M+NH4]+ |
| SQDG 16:0_18:2 | C43H78O12S | 15.52 | [M-H]- | [M+NH4]+ |
| SQDG 16:0_18:3 | C43H76O12S | 14.96 | [M-H]- | [M+NH4]+ |
| SQDG 18:2_18:2 | C45H78O12S | 15.04 | - | [M+NH4]+ |
| SQDG 18:2_18:3 | C45H76O12S | 14.42 | [M-H]- | [M+NH4]+ |
| acMGDG 52:3 | C61H110O11 | 20.51 | [M+CHO2]- | - |
| acMGDG 52:4 | C61H108O11 | 20.21 | [M+CHO2]- | - |
| acMGDG 54:4 | C63H112O11 | 20.56 | [M+CHO2]- | - |
| acMGDG 54:5 | C63H110O11 | 20.26 | [M+CHO2]- | - |
| acMGDG 18:2_18:2_18:2 | C63H108O11 | 19.93 | [M+CHO2]- | [M+NH4]+ |
| acMGDG 18:2_18:2_18:3 | C63H106O11 | 19.62 | [M+CHO2]- | [M+NH4]+ |
| acMGDG 18:2_18:3_18:3 | C63H104O11 | 19.3 | - | [M+NH4]+ |
| acMGDG 18:3_18:3_18:3 | C63H102O11 | 18.93 | - | [M+NH4]+ |
| DG 16:0_16:0 | C35H68O5 | 18.52 | - | [M+NH4]+ |
| DG 16:0_16:1 / DG 14:0_18:1 | C35H66O5 | 17.91 | - | [M+NH4]+ |
| DG 16:0_18:1 | C37H70O5 | 18.58 | - | [M+NH4]+ |
| DG 16:0_18:2 | C37H68O5 | 18.13 | - | [M+NH4]+ |
| DG 16:1_18:2 | C37H66O5 | 17.42 | - | [M+NH4]+ |
| DG 16:0_18:3 | C37H66O5 | 17.59 | - | [M+NH4]+ |
| DG 16:2_18:2 | C37H64O5 | 16.89 | - | [M+NH4]+ |
| DG 17:0_18:2 | C38H70O5 | 18.4 | - | [M+NH4]+ |
| DG 18:0_18:1 | C39H74O5 | 19.2 | - | [M+NH4]+ |
| DG 18:1_18:1 | C39H72O5 | 18.66 | - | [M+NH4]+ |
| DG 18:0_18:2 | C39H72O5 | 18.73 | - | [M+NH4]+ |
| DG 18:1_18:2 | C39H70O5 | 18.14 | - | [M+NH4]+ |
| DG 18:0_18:3 | C39H70O5 | 18.31 | - | [M+NH4]+ |
| DG 18:2_18:2 | C39H68O5 | 17.61 | - | [M+NH4]+ |
| DG 18:2_18:3 | C39H66O5 | 17.09 | - | [M+NH4]+ |
| DG 18:2_19:1 | C40H72O5 | 18.44 | - | [M+NH4]+ |
| DG 18:2_20:1 | C41H74O5 | 18.75 | - | [M+NH4]+ |
| DG 18:2_22:0 | C43H80O5 | 19.87 | - | [M+NH4]+ |
| TG 12:0_16:0_18:1 / TG 14:0_16:0_16:1 / TG 14:0_14:0_18:1 | C49H92O6 | 21.12 | - | [M+NH4]+ |
| TG 12:0_16:1_18:1 / TG 12:0_16:0_18:2 / TG 14:0_14:0_18:2 / TG 14:0_16:1_16:1 | C49H90O6 | 20.8 | - | [M+NH4]+ |
| TG 10:1_18:1_18:1 / TG 14:1_14:1_18:1 / TG 12:1_16:1_18:1 / TG 12:0_16:0_18:3 / TG 12:1_16:0_18:2 / TG 14:0_14:0_18:3 / TG 14:0_14:1_18:2 / TG 12:0_16:1_18:2 / TG 14:1_16:1_16:1 | C49H88O6 | 20.57 | - | [M+NH4]+ |
| TG 16:0_16:0_16:0 | C51H98O6 | 21.76 | - | [M+NH4]+ |
| TG 14:0_16:0_18:1 / TG 16:0_16:0_16:1 | C51H96O6 | 21.45 | - | [M+NH4]+ |
| TG 14:0_16:0_18:2 / TG 14:0_16:1_18:1 / TG 16:0_16:1_16:1 / TG 12:0_18:1_18:1 | C51H94O6 | 21.16 | - | [M+NH4]+ |
| TG 14:0_16:0_18:3 / TG 14:0_16:1_18:2 / TG 14:1_16:0_18:2 / TG 16:1_16:1_16:1 | C51H92O6 | 20.9 | - | [M+NH4]+ |
| TG 12:0_18:2_18:2 | C51H90O6 | 20.52 | - | [M+NH4]+ |

| | | | | |
|---|---|---|---|---|
| TG 49:2 | C52H96O6 | 21.42 | - | [M+NH4]+ |
| TG 15:1_16:0_18:2 | C52H94O6 | 21.11 | - | [M+NH4]+ |
| TG 16:0_16:0_18:1 | C53H100O6 | 21.77 | - | [M+NH4]+ |
| TG 16:0_16:1_18:1 / TG 16:0_16:0_18:2 | C53H98O6 | 21.49 | - | [M+NH4]+ |
| TG 16:1_16:1_18:1 / TG 16:0_16:1_18:2 / TG 14:0_18:1_18:2 | C53H96O6 | 21.23 | - | [M+NH4]+ |
| TG 14:0_18:2_18:2 / TG 16:1_16:1_18:2 | C53H94O6 | 20.9 | - | [M+NH4]+ |
| TG 14:0_18:2_18:3 / TG 14:1_18:2_18:2 | C53H92O6 | 20.61 | - | [M+NH4]+ |
| TG 14:2_18:2_18:2 / TG 14:0_18:3_18:3 | C53H90O6 | 20.33 | - | [M+NH4]+ |
| TG 16:0_17:0_18:2 / TG 16:0_17:1_18:1 / TG 17:0_17:1_17:1 / TG 15:0_18:1_18:1 | C54H100O6 | 21.66 | - | [M+NH4]+ |
| TG 15:1_18:1_18:1 / TG 16:0_17:2_18:1 / TG 16:0_17:1_18:2 / TG 15:0_18:1_18:2 / TG 17:1_17:1_17:1 | C54H98O6 | 21.39 | - | [M+NH4]+ |
| TG 15:1_18:2_18:2 | C54H94O6 | 20.83 | - | [M+NH4]+ |
| TG 15:1_18:2_18:3 | C54H92O6 | 20.54 | - | [M+NH4]+ |
| TG 16:0_18:0_18:0 / TG 16:0_16:0_20:0 | C55H106O6 | 22.33 | - | [M+NH4]+ |
| TG 16:0_18:0_18:1 | C55H104O6 | 22.06 | - | [M+NH4]+ |
| TG 16:0_18:1_18:1 | C55H102O6 | 21.79 | - | [M+NH4]+ |
| TG 16:0_18:1_18:2 / TG 16:1_18:1_18:1 | C55H100O6 | 21.52 | - | [M+NH4]+ |
| TG 16:0_18:2_18:2 | C55H98O6 | 21.25 | - | [M+NH4]+ |
| TG 16:0_18:2_18:3 / TG 16:1_18:2_18:2 | C55H96O6 | 20.99 | - | [M+NH4]+ |
| TG 16:0_18:3_18:3 / TG 16:1_18:2_18:3 | C55H94O6 | 20.71 | - | [M+NH4]+ |
| TG 16:1_18:3_18:3 | C55H92O6 | 20.37 | - | [M+NH4]+ |
| TG 17:0_18:1_18:1 / TG 17:0_18:0_18:2 / TG 16:0_18:2_19:0 | C56H104O6 | 21.95 | - | [M+NH4]+ |
| TG 17:0_18:1_18:2 / TG 17:1_18:1_18:1 | C56H102O6 | 21.68 | - | [M+NH4]+ |
| TG 17:0_18:2_18:2 | C56H100O6 | 21.42 | - | [M+NH4]+ |
| TG 17:1_18:2_18:2 | C56H98O6 | 21.14 | - | [M+NH4]+ |
| TG 17:2_18:2_18:2 | C56H96O6 | 20.81 | - | [M+NH4]+ |
| TG 17:2_18:2_18:3 | C56H94O6 | 20.54 | - | [M+NH4]+ |
| TG 16:0_18:0_20:0 | C57H110O6 | 22.58 | - | [M+NH4]+ |
| TG 16:1_18:0_20:0 / TG 16:0_18:1_20:0 | C57H108O6 | 22.33 | - | [M+NH4]+ |
| TG 18:0_18:1_18:1 / TG 18:0_18:0_18:2 | C57H106O6 | 22.08 | - | [M+NH4]+ |
| TG 18:1_18:1_18:1 / 18:0_18:1_18:2 | C57H104O6 | 21.81 | - | [M+NH4]+ |
| TG 18:1_18:1_18:2 | C57H102O6 | 21.57 | - | [M+NH4]+ |
| TG 18:1_18:2_18:2 | C57H100O6 | 21.27 | - | [M+NH4]+ |
| TG 18:1_18:2_18:3 | C57H98O6 | 20.98 | - | [M+NH4]+ |
| TG 18:2_18:2_18:3 | C57H96O6 | 20.71 | - | [M+NH4]+ |
| TG 18:2_18:3_18:3 | C57H94O6 | 20.43 | - | [M+NH4]+ |
| TG 18:3_18:3_18:3 | C57H92O6 | 20.13 | - | [M+NH4]+ |
| TG 16:0_18:2_21:0 / TG 18:1_18:1_19:0 | C58H108O6 | 22.23 | - | [M+NH4]+ |
| TG 18:1_18:2_19:0 / TG 18:0_18:2_19:1 / TG 18:0_18:3_19:0 / TG 18:1_18:1_19:1 / TG 16:0_18:3_21:0 | C58H106O6 | 22 | - | [M+NH4]+ |
| TG 18:2_18:2_19:0 | C58H104O6 | 21.72 | - | [M+NH4]+ |
| TG 18:2_18:2_19:2 | C58H100O6 | 21.16 | - | [M+NH4]+ |

| | | | | |
|---|---|---|---|---|
| TG 16:0_18:1_22:0 / TG 18:0_18:1_20:0 | C59H112O6 | 22.57 | - | [M+NH4]+ |
| TG 18:1_18:1_20:0 | C59H110O6 | 22.35 | - | [M+NH4]+ |
| TG 18:1_18:2_20:0 / TG 18:0_18:3_20:0 | C59H108O6 | 22.13 | - | [M+NH4]+ |
| TG 18:2_18:2_20:0 | C59H106O6 | 21.88 | - | [M+NH4]+ |
| TG 18:2_18:3_20:0 | C59H104O6 | 21.62 | - | [M+NH4]+ |
| TG 18:3_18:3_20:0 | C59H102O6 | 21.32 | - | [M+NH4]+ |
| TG 16:0_18:1_23:0 | C60H114O6 | 22.68 | - | [M+NH4]+ |
| TG 18:1_18:1_21:0 / TG 16:0_18:2_23:0 / TG 18:0_18:2_21:0 | C60H112O6 | 22.48 | - | [M+NH4]+ |
| TG 18:1_18:2_21:0 | C60H110O6 | 22.27 | - | [M+NH4]+ |
| TG 18:2_18:2_21:0 | C60H108O6 | 22.02 | - | [M+NH4]+ |
| TG 16:0_18:1_24:0 / TG 18:0_18:1_22:0 | C61H116O6 | 22.8 | - | [M+NH4]+ |
| TG 18:1_18:1_22:0 | C61H114O6 | 22.58 | - | [M+NH4]+ |
| TG 18:1_18:2_22:0 / TG 18:0_18:3_22:0 | C61H112O6 | 22.38 | - | [M+NH4]+ |
| TG 18:2_18:2_22:0 / TG 18:1_18:3_22:0 | C61H110O6 | 22.16 | - | [M+NH4]+ |
| TG 18:2_18:3_22:0 | C61H108O6 | 21.95 | - | [M+NH4]+ |
| TG 18:3_18:3_22:0 | C61H106O6 | 21.73 | - | [M+NH4]+ |
| TG 18:1_18:1_23:0 | C62H116O6 | 22.69 | - | [M+NH4]+ |
| TG 18:1_18:2_23:0 | C62H114O6 | 22.49 | - | [M+NH4]+ |
| TG 18:2_18:2_23:0 | C62H112O6 | 22.29 | - | [M+NH4]+ |
| TG 18:1_18:1_24:0 | C63H118O6 | 22.81 | - | [M+NH4]+ |
| TG 18:1_18:2_24:0 | C63H116O6 | 22.61 | - | [M+NH4]+ |
| TG 18:2_18:2_24:0 / TG 18:1_18:3_24:0 | C63H114O6 | 22.41 | - | [M+NH4]+ |
| TG 18:2_18:3_24:0 | C63H112O6 | 22.22 | - | [M+NH4]+ |
| TG 18:3_18:3_24:0 | C63H110O6 | 22.02 | - | [M+NH4]+ |
| TG 18:1_18:1_25:0 | C64H120O6 | 22.9 | - | [M+NH4]+ |
| TG 18:2_18:2_25:0 | C64H116O6 | 22.52 | - | [M+NH4]+ |
| TG 18:1_18:1_26:0 / TG 18:0_18:2_26:0 | C65H122O6 | 23.01 | - | [M+NH4]+ |
| TG 18:2_18:2_26:0 | C65H118O6 | 22.64 | - | [M+NH4]+ |
| ST 18:1 | C47H82O2 | 22.21 | - | [M+NH4]+ |
| ST 18:2 | C47H80O2 | 21.89 | - | [M+NH4]+ |
| ST 18:3 | C47H78O2 | 21.61 | - | [M+NH4]+ |
| Glc-ST 14:0 | C49H86O7 | 19.31 | - | [M+NH4]+ |
| Glc-ST 15:0 | C50H88O7 | 19.65 | [M+CHO2]- | [M+NH4]+ |
| Glc-ST 16:0 | C51H90O7 | 19.82 | [M+CHO2]- | [M+NH4]+ |
| Glc-ST 16:1 | C51H88O7 | 19.53 | [M+CHO2]- | [M+NH4]+ |
| Glc-ST 17:0 | C52H92O7 | 20.09 | [M+CHO2]- | [M+NH4]+ |
| Glc-ST 17:1 | C52H90O7 | 19.71 | [M+CHO2]- | - |
| Glc-ST 17:2 | C52H88O7 | 19.31 | [M+CHO2]- | [M+NH4]+ |
| Glc-ST 17:3 | C52H86O7 | 18.92 | - | [M+NH4]+ |
| Glc-ST 18:0 | C53H94O7 | 20.28 | [M+CHO2]- | [M+NH4]+ |
| Glc-ST 18:1 | C53H92O7 | 19.8 | [M+CHO2]- | [M+NH4]+ |
| Glc-ST 18:2 | C53H90O7 | 19.49 | [M+CHO2]- | [M+NH4]+ |
| Glc-ST 18:3 | C53H88O7 | 19.14 | [M+CHO2]- | [M+NH4]+ |
| Glc-ST 22:0 | C57H102O7 | 21.1 | [M+CHO2]- | [M+NH4]+ |
| Glc-ST 23:0 | C58H104O7 | 21.26 | [M+CHO2]- | - |
| Glc-ST 24:0 | C59H106O7 | 21.47 | [M+CHO2]- | - |

**Table S4** – For each polarity, the top 10 MST connections with the lowest distance (highest spectral similarity) created with the biological dataset of grape lipidomics, processed with the tailored denoising method. The shown connections are relative to the leftmost part of the MST plots (blue line in Figure S5). All the connections are relative to similar compounds belonging to the same classes (first letters of compound names are relative to compound class: Glc-ST = hexosyl sitosterol ester, PA = Phosphatidic

acid, PC = Phosphatidylcholine, PE = Phosphatidylethanolamine, PI = Phosphatidylinositol, TG = Triglyceride). The score differences between the similarity score of involved compounds before and after applying the tailored denoising approach are listed. All of them are positive, indicating higher similarity scores for denoised data.

| **Positive** | | |
|---|---|---|
| from | to | similarity improvement |
| TG 16:0_18:1_18:1 | TG 18:1_18:1_20:0 | 0.12198809 |
| TG 18:2_18:2_23:0 | TG 18:2_18:2_25:0 | 0.10288792 |
| TG 18:1_18:2_18:2 | TG 18:2_18:2_23:0 | 0.07801753 |
| TG 18:1_18:1_24:0 | TG 18:1_18:1_20:0 | 0.11354113 |
| TG 18:3_18:3_22:0 | TG 18:3_18:3_20:0 | 0.12328372 |
| TG 18:2_18:3_20:0 | TG 18:2_18:3_24:0 | 0.08079547 |
| TG 16:0_18:1_18:2 / TG 16:1_18:1_18:1 | TG 18:1_18:2_20:0 / TG 18:0_18:3_20:0 | 0.05775242 |
| TG 18:2_18:2_19:2 | TG 17:2_18:2_18:2 | 0.07929233 |
| PC 33:2 | PC 17:0_18:2 | 0.05760571 |
| PC 18:1_18:1 | PC 17:0_18:2 | 0.0879109 |
| **Negative** | | |
| from | to | similarity improvement |
| PI 16:0_18:2 | PI 16:0_18:3 | 0.231498884 |
| Glc-ST 24:0 | Glc-ST 17:1 | 0.253217906 |
| PE 18:2_20:0 | PE 18:2_24:0 | 0.370198509 |
| PC 16:0_18:3 | PC 18:2_18:2 | 0.074558579 |
| PC 16:0_18:2 | PC 18:2_18:2 | 0.084660304 |
| PC 18:2_18:2 | PC 18:0_18:1 | 0.222677434 |
| PC 16:0_18:2 | PC 18:2_20:0 | 0.124571124 |
| PC 18:2_20:0 | PC 18:3_18:3 | 0.168040195 |
| PA 16:0_18:2 | PA 16:0_18:3 | 0.042070435 |
| PC 18:1_18:2 | PC 18:2_20:0 | 0.118913591 |

**Figure S1 -** Distribution of the increase in zero values in the cleaned data similarity matrix (with 5% base ions intensity cutoff method) based on the chemical Tanimoto similarity of the compounds involved in the comparisons for the in-house standard library dataset.

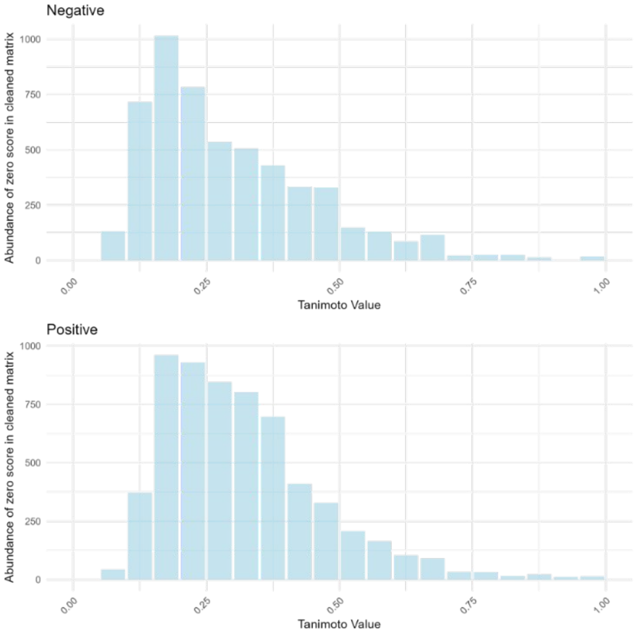
Negative
Abundance of zero score in cleaned matrix
1000
750
500
250
0
0.00
0.25
0.50
0.75
1.00
Tanimoto Value
Positive
Abundance of zero score in cleaned matrix
1000
750
500
250
0
0.00
0.25
0.50
0.75
1.00
Tanimoto Value

**Figure S2 –** Ordered intensity behaviour of randomly selected MS/MS spectra from ToF and Orbitrap GNPS datasets from MassBank of North America (MoNA) repository used for MNs and MST creation. The title of each spectra is its corresponding ID. The behaviour of ions' ordered intensity discussed in Figure 1 is generally observed.

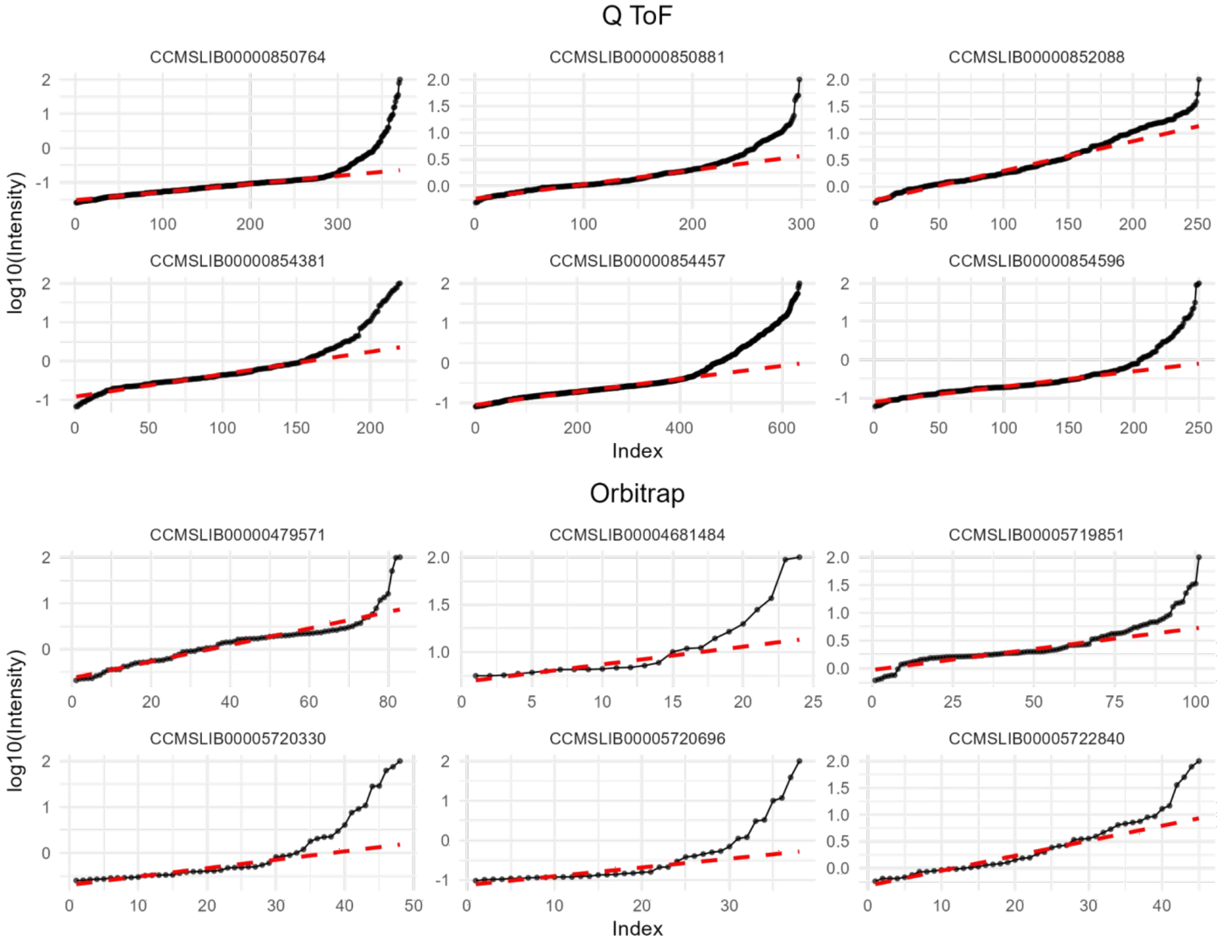

**Figure S3** – MST of all different standards datasets analysed with a focus on the leftmost part (higher similarity values)

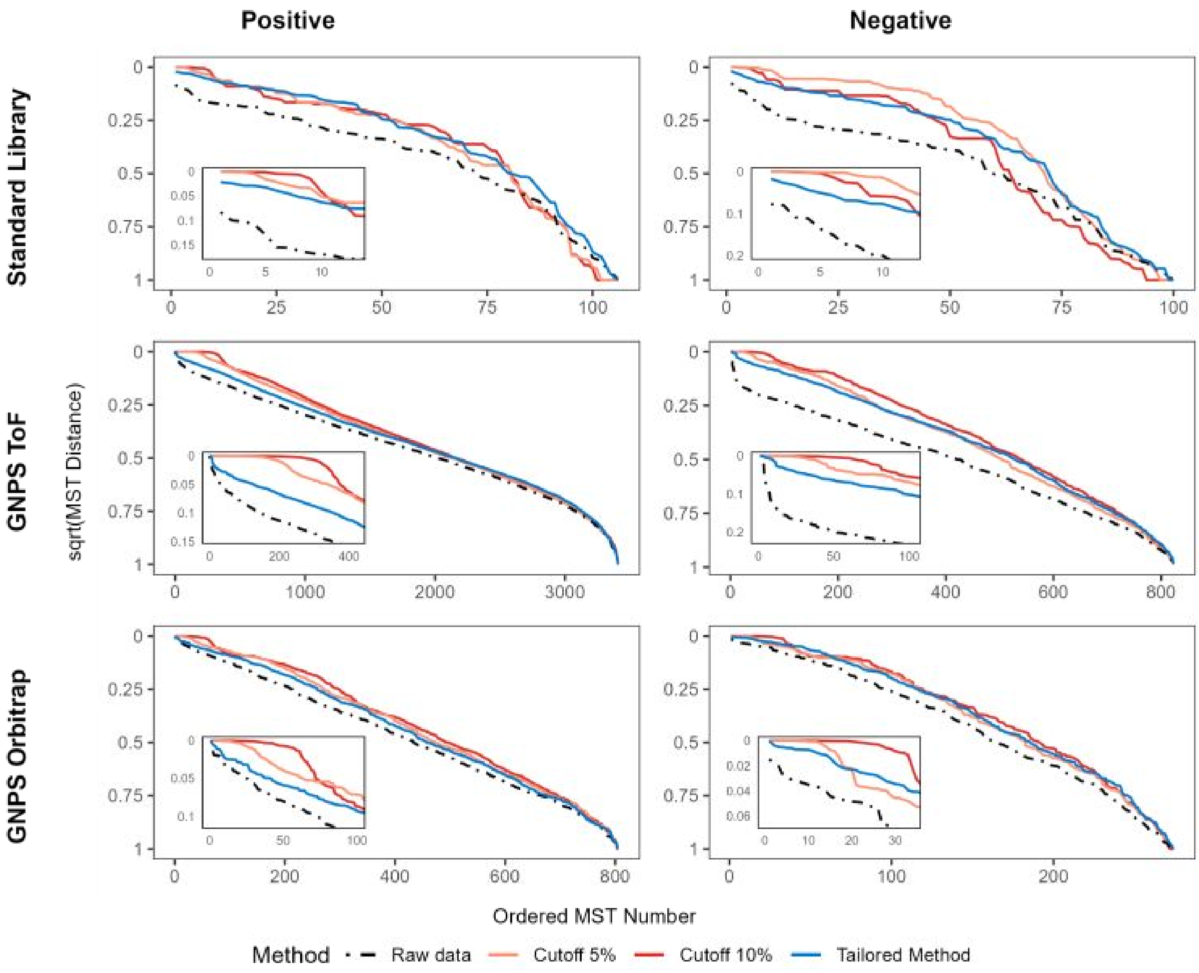

**Figure S4** – Ordered MST distance plot of MNs created with different denoising approaches of biological sample data originated from a lipidomic study on grapes, positive and negative ionization modes (data from Table S3).

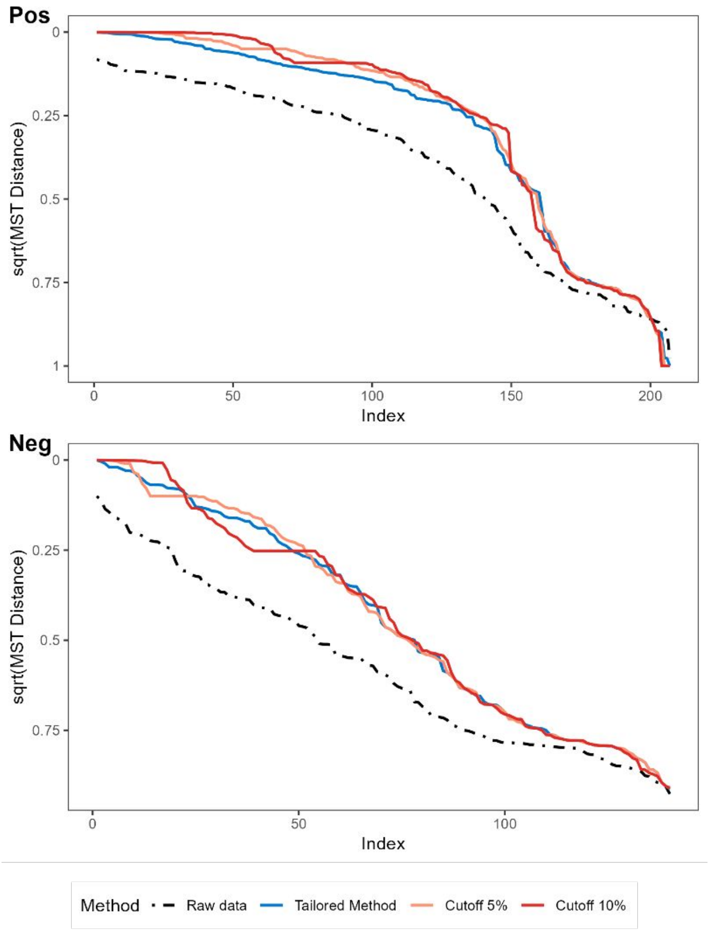


**Figure S5** – Number of zero distances (<0.001) in MST according to different denoising cut-off percentages. Data from ToF dataset, positive ionization mode. The number of zeros increase while the median of the lowest 5$^{th}$ percentile of MST distances decreases.

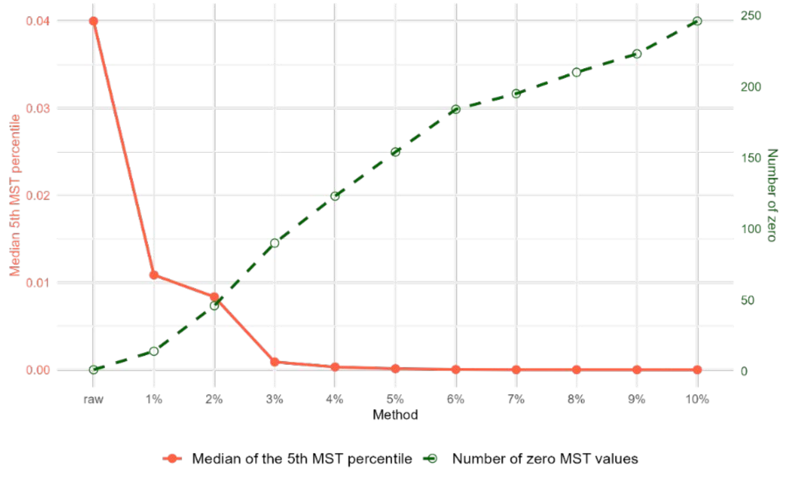

**Figure S6** – Median values of the 5th percentile of ordered MST similarities and corresponding number of ions removed, plotted against increasing denoising severity for all datasets and ionization polarities analyzed. The trends show a consistent decrease in MST similarity and an increase in ion loss with higher cutoff thresholds across datasets, although some variability is observed in the specific cutoff percentage at which MST similarity values approach zero.

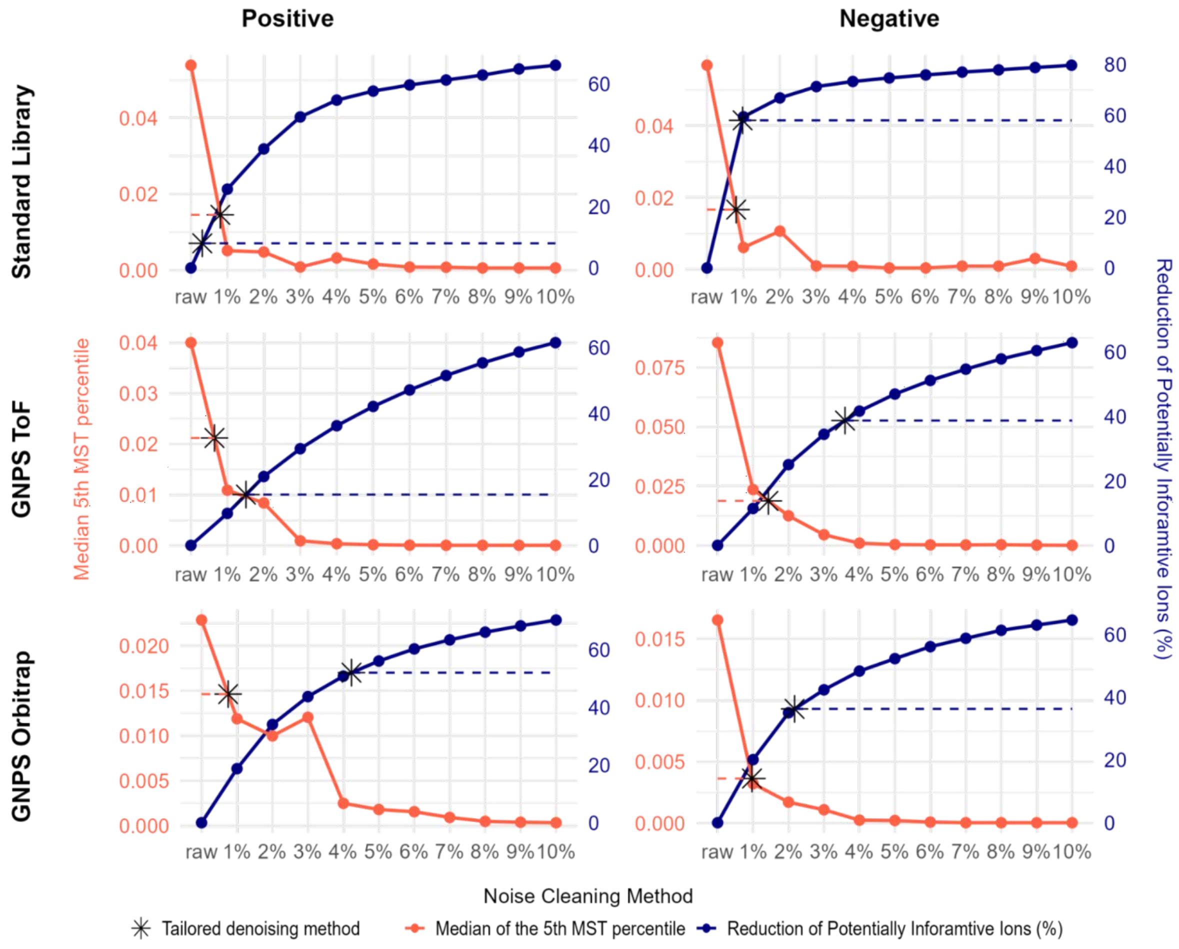

Supplement: Supplementary file 1 [file ac5c02109_si_001.pdf]
